# Over-dispersion in malaria–schistosoma co-infection: insights from a meta-analytical approach and systematic review

**DOI:** 10.1186/s40249-026-01490-1

**Published:** 2026-10-09

**Authors:** Célia Koellsch–Amet, Jérôme Boissier, Ronaldo Augusto

**Affiliations:** IHPE, University of Perpignan Via Domitia, CNRS, Ifremer, University of Montpellier, Perpignan, France

## Abstract

**Background:**

Malaria and schistosomiasis are two major parasitic diseases that are co-endemic in many regions of sub-Saharan Africa. Despite their frequent geographical overlap, the potential epidemiological patterns and potential interactions associated with *Plasmodium*–-*Schistosoma* co-infection remain poorly characterized. This study aimed to systematically assess the epidemiology of malaria–schistosomiasis co-infection and to quantify whether co-infections occur more frequently than expected under an assumption of independence between the two infections.

**Methods:**

We conducted a systematic review and meta-analysis in accordance with the PRISMA 2020 guidelines. A total of 192 studies published between 1996 and 2023 were included in the qualitative synthesis, of which 59 studies involving 73,383 individuals were included in the meta-analysis. Pooled odds ratio (*ORs*) were estimated to compare observed and expected co-infection prevalence.

**Results:**

The pooled analysis showed that *Plasmodium-Schistosoma* co-infection occurs more frequently than expected by chance (overall *OR* = 1.20, 95% *CI* 1.02–1.40), despite substantial heterogeneity across studies (*I*^*2*^ = 89%). Reported co-infection prevalence ranged from 1.1% in Benin to 36.6% in Mali. School-age children and pregnant women were disproportionately affected. Subgroup analyses revealed no consistent differences according to sex or parasite species.

**Conclusions:**

These findings indicate a non-random distribution of malaria-schistosomiasis co-infection in sub-Saharan Africa, consistent with epidemiological over-dispersion. Although the underlying biological and immunological mechanisms remain uncertain, the widespread co-occurrence of these parasites highlights the importance of integrated surveillance and control strategies that account for polyparasitism in endemic settings.

**Supplementary Information:**

The online version contains supplementary material available at https://doi.org/10.1186/s40249-026-01490-1.

## Background

Malaria and schistosomiasis are among the most widespread and invalidating parasitic diseases affecting 85 and 78 countries, respectively [1, 2]. On a global scale, it is estimated that 46.4 million DALYs (Disability-Adjusted Life Years) were lost due to malaria [3], compared with 1.6 million for schistosomiasis [4], making these two parasitic diseases the two most important in the world; More than a third of the world's population is thought to be infected by helminths or *Plasmodium* [5] with Africa remaining the hardest-hit continent. Sub-Saharan Africa bears the greatest burden of both infections, where overlapping ecological and socio-economic conditions create extensive zones of co-endemicity [6–10]. Moreover, the epidemiology of co-infection is influenced by a variety of factors including population dynamics, behavioural, genetic, host physio-immunology, population size, and parasite dispersal patterns [6]. These include the high frequency of the two parasites in the same population, the similar geographical distribution of the vectors and the need for an aquatic environment to complete both parasite life cycles. Whether *P lasmodium* or schistosome, the two parasites have complex life cycles, dependent on environmental conditions that allow other host species—mosquitoes for *Plasmodium*, freshwater snails for *Schistosoma*—to thrive. Furthermore, spatial statistical models highlight the geographical overlap and thus support the co-endemicity of malaria and schistosomiasis infections [11]. Environmental sensitivity and underprivileged socio-economic conditions have repeatedly been reported to influence the survival of free-living invertebrate hosts and helminths, thereby governing the spatial distribution of diseases and favouring the geographical overlap of *Plasmodium* and *Schistosoma* [7, 12]. According to recent global estimates, malaria and schistosomiasis are co-endemic in 51 countries worldwide, with the highest overlap observed in sub-Saharan Africa [13, 14]. Although malaria and schistosomiasis are co-endemic in several regions worldwide, the available literature on *Plasmodium*–-*Schistosoma* co-infection is overwhelmingly concentrated in sub-Saharan Africa, which consequently represents the primary geographical focus of this review.Since the last major pandemic of COVID-19 that recently affected the world, the question of an interaction between climate change and infectious diseases has become increasingly worrying [15]. As the effects of these changes are still poorly understood, climate change as a whole is generating major uncertainties about the epidemiology of many diseases, and is widening the gap between current healthcare techniques and the evolution of the epidemiology of infectious diseases. To date, it is estimated that 58% of infectious diseases in humans have at some time been intensified by climatic events [16]. Climatic factors can also lead to the emergence of new pathogens and increase the risk of transmission of existing diseases such as malaria and other neglected tropical and subtropical diseases such as schistosomiasis [17–19]. In Africa, malaria episodes are impacted by environmental variability and often occur after climatic anomalies such as periods of drought or extreme rainfall [20, 21]. However, falciparum malaria remains confined to tropical and subtropical regions in the various predictive models [22], while *Plasmodium vivax*, formerly the dominant species in Europe, could re-emerge [23, 24]. An outbreak of schistosomiasis has also recently been identified in southern Europe (Corsica, France), with infected patients originated from France, Germany and Italy [25, 26], resulting in both of an increase in the human migration and of the rise in the temperature of fresh water ecosystems, which favours the establishment of the snail vector in southern Europe [27]. Identifying current hotspots of co-endemicity and understanding the ecological drivers of overlap will be critical for anticipating future shifts in disease distribution. Although the epidemiology and immunology of major parasitic diseases such as malaria and schistosomiasis are relatively well documented, the mechanisms and implications of concomitant infections remain largely unexplored. However, co-infection with *Plasmodium* spp. and *Schistosoma* spp. is common in many endemic regions and could be modulated by global warming, prompting growing interest in understanding the extent to which these parasites can interact in the same host [19, 28]. In particular, several studies suggest that *Schistosoma* infections may modulate susceptibility or immune response to *Plasmodium* infection, raising key questions about the potential interactions between these pathogens, both clinically and immunologically. Interactions between *Plasmodium* and helminths have already been demonstrated to influence the host immune response to malaria infection, as helminth-induced Th2-biased and regulatory immune responses may alter the Th1-dependent mechanisms involved in malaria control [29, 30]. In the specific case of schistosomiasis, several studies also suggest the existence of cross-effects with malaria, although the exact nature of these interactions—protective or deleterious—remains controversial [31–40]. Some studies report that co-infection with schistosomes could worsen *Plasmodium* infection, by increasing the intensity, prevalence or clinical impact of *Plasmodium* spp. [31, 34, 38, 41, 42]. Conversely, other studies indicate a mitigating or even protective effect of schistosomiasis on the severity of malaria [32, 37, 40, 42]. At the same time, some studies have found no significant association between these two infections [43–46]. These controversies may stem from the fact that sampling protocols vary between studies. The strains of parasites studied may also differ from one study to another, as may the sampling region. The timing of infection could influence the outcome of co-infection, whether simultaneous or sequential and this still is an aspect neglected in epidemiological analyses.

Observational and experimental studies, in both animals and humans, have begun to reveal the mechanisms of these interactions, whether direct (through competition for resources) or indirect (via modulation of the immune response). Nevertheless, the complexity of interactions between *Plasmodium* spp. and *Schistosoma* spp. continues to be investigated, with the aim of better defining the synergistic or antagonistic effects of these co-infections in human populations [47–49]. To date, the appearance of simultaneous infections by these two parasites in humans seems to be at the origin of the emergence of several studies aimed at elucidating aspects related to the synergistic and antagonistic interactions of *Plasmodium* spp. and *Schistosoma* spp. [47–49].

Despite growing interest in malaria-helminth co-infections, the epidemiology of *Plasmodium*–-*Schistosoma* co-infection remains fragmented, and it is unclear whether these infections co-occur randomly or follow non-independent patterns at the population level. In this study, we conducted a systematic review and meta-analysis to estimate the prevalence of malaria–schistosomiasis co-infection and to test the hypothesis that co-infections occur more frequently than expected under an assumption of independence, consistent with epidemiological over-dispersion.

The primary objectives of this study were to estimate the prevalence of malaria–schistosomiasis co-infection and to assess whether the observed frequency of co-infection differed from that expected under an assumption of independence between the two infections. Accordingly, the interaction examined in this study was epidemiological in nature and did not aim to infer causal or mechanistic relationships.

## Method

### Protocol and registration

This systematic review and meta-analysis was conducted in accordance with the Preferred Reporting Items for Systematic Reviews and Meta-Analyses (PRISMA 2020) guidelines. The PRISMA 2020 checklist is provided in Appendix S1. The aim of the approach was to explore and synthesise the available data on interactions between Plasmodium spp. and Schistosoma spp. in contexts of human co-infection.

### Information sources and search strategy

Literature searches were conducted on 24 January 2025 in PubMed and BibCNRS, which provide comprehensive coverage of biomedical and life sciences literature. Additional databases were not included due to substantial overlap in indexed journals and to ensure feasibility and consistency of study screening. We recognize that this choice may have resulted in the omission of some relevant studies indexed exclusively in other databases (e.g. Scopus or Web of Science), and thus represents a potential source of selection bias. To mitigate this limitation, database searches were complemented by citation tracking and manual grey literature screening. The search strategy combined the following terms using Boolean operators: (“Plasmodium” OR “malaria”) AND (“Schistosoma” OR “schistosomiasis”) AND (“co-infection” OR “coinfection”). This search was completed by a citation search of articles included between 28 January and 6 March 2025.

Where article abstracts were not available, a selection was made from the tables of contents, followed by a full-text analysis. In addition, a manual grey literature search was conducted and managed using Microsoft Excel 2016 and Zotero 7.0.32 software (Vienna, VA: Corporation for Digital Scholarship, 2025).

### Eligibility criteria

Studies were included if they (1) reported original epidemiological data on malaria and schistosomiasis co-infection, (2) were conducted in human populations, (3) provided sufficient data to estimate co-infection prevalence, and (4) were published in English or French. Studies were excluded if they were reviews, case reports, experimental laboratory studies, or if they did not report extractable data on co-infection prevalence. Studies were excluded if they were reviews, case reports, experimental laboratory studies, studies focusing solely on the treatment of infections, studies mentioning co-infections with geohelminths without specific data on *Schistosoma*, unpublished studies, protocols, grey literature that was not accessible, or if they did not report usable data on the prevalence of co-infections.

This analysis did not distinguish between asymptomatic and uncomplicated malaria, nor between the degrees of severity of the two parasitic diseases, in order to estimate the nature of the association between co-infection with *Schistosoma* and *Plasmodium*.

Although no geographical restrictions were applied during the literature search, all studies meeting the inclusion criteria were conducted in Africa, reflecting the current distribution of published epidemiological data on malaria–schistosomiasis co-infection.

### Information and data extraction

The data were extracted into a dedicated Excel sheet, including the following information for each study: References (author, year); Period and location of study; Gender and age range of the population; Total number of participants and number of cases for each group (co-infected, mono-infected, uninfected); Prevalence rates for Plasmodium spp., Schistosoma spp. and co-infection; Additional information: anaemia, malnutrition, other associated pathologies.

These data were then imported into R version 4.3.3 (Vienna: R Foundation for Statistical Computing, 2024) via CSV files. The articles were classified by type: epidemiological surveys, functional studies and experimental studies. Epidemiological studies are subjected to a meta-analysis, while other studies are subjected to a written synthesis. Using this spreadsheet, a preliminary summary of the results of the included studies was developed. Relationships within and between studies were also examined, as was the robustness of the synthesis for each study. The articles were grouped by type of study under three headings: epidemiological investigations, functional investigations and experimental studies. Each of these headings represents a paragraph of this review.

### Statistical analysis

Concerning epidemiological studies, in the Excel sheet of the included studies, the percentages of expected and observed co-infection were calculated (Appendix S2).

- Observed co-infection (actual co-infection measured in the study):

This proportion is calculated from the number of people co-infected (infected with both *Plasmodium* and *Schistosoma*) in relation to the total sample size.

$$Observed\, coinfection \left( \% \right) = \left( {\frac{Number\, of\, coinfected}{{Total\, sample\, size}}} \right) \times 100$$

- Expected co-infection (if the infections were independent):

This is a theoretical estimate of the co-infection that would occur if *Plasmodium* and *Schistosoma* infections were independently distributed in the population. It is calculated by multiplying the individual prevalence of each parasite. Two events are independent when the occurrence of one does not influence the probability of the other. In this case, the probability of both events occurring is the product of their probabilities.

$$P\left( {A \cap B} \right) = P\left( A \right) \times P\left( B \right)$$

P(A∩B) is the expected prevalence of parasite co-infection under the null hypothesis of the absence parasite interaction.

A is the prevalence of schistosome infection (Number of *S chistosoma* infected people/Total). B is the prevalence of *P lasmodium* infection (Number of *P lasmodium* infected people/Total):

$$Expected\, coinfection \left( \% \right)\, = \,\left( {\frac{Nb\, of\, Plasmodium\, infected}{{Total}} \times \frac{Nb\, of\, Schistosoma\, infected}{{Total}}} \right) \times 100$$

A Fisher's test was performed to compare the expected and observed co-infection rates.The statistical analysis was then carried out in R using the metafor and meta packages. Meta-analyses were performed on the odds ratios (*OR*) using the Mantel–Haenszel method, with fixed-effects and random-effects models. Several sub-analyses were conducted: By type of co-infection (*S. mansoni* vs. *S. haematobium*/*P. falciparum* vs. *P.vivax*); By sex (men vs. women); By overall prevalence. An *OR* > 1 suggests that co-infection is more frequent than expected by chance and suggests a positive association and an *OR* < 1 suggests that co-infection is less frequent than expected, so a potential protective effect. Heterogeneity between studies was assessed using the I^2^ and Tau^2^ indicators. Forest plots were generated to visualise the results, and the presence of publication bias was analysed by funnel plot. This bias was also tested more formally using the Egger asymmetry test (metabias). A Fisher's exact test was used to assess the association between certain categorical variables on a point-by-point basis.

### Assessment of study quality and risk of bias

The methodological quality of the studies included in the meta-analysis was assessed using the Joanna Briggs Institute (JBI) Critical Appraisal Checklist for Studies Reporting Prevalence Data. This tool evaluates the risk of bias across nine domains, including the appropriateness of the sampling frame, recruitment methods, sample size adequacy, description of study subjects and setting, data analysis coverage, validity and reliability of the methods used to identify the condition, consistency of measurement across participants, appropriateness of statistical analyses, and adequacy of the response rate. Each item was rated as “Yes”, “No”, “Unclear”, or “Not applicable”. An overall quality score was then calculated as the proportion of “Yes” responses among applicable items. Studies were subsequently classified as low risk of bias (≥ 71% “Yes”), moderate risk of bias (50–70%), or high risk of bias (< 50%) (Supp. Table 1).

### Co-occurrence map

To depict global patterns of malaria–schistosomiasis overlap, we compiled country-level data on malaria incidence and schistosomiasis occurrence from the World Health Organization Global Health Observatory and the Preventive Chemotherapy and Transmission Control (PCT) Databank (data accessed January 2025). Annual records from 2017–2023 were aggregated to classify each country as malaria only, schistosomiasis only, co-occurrence, or none. Maps were generated in R version 4.3.3 (Vienna: R Foundation for Statistical Computing, 2024) using the *ggplot2* and *choroplethr* packages.

## Results

### Search results

**Fig. 1:**
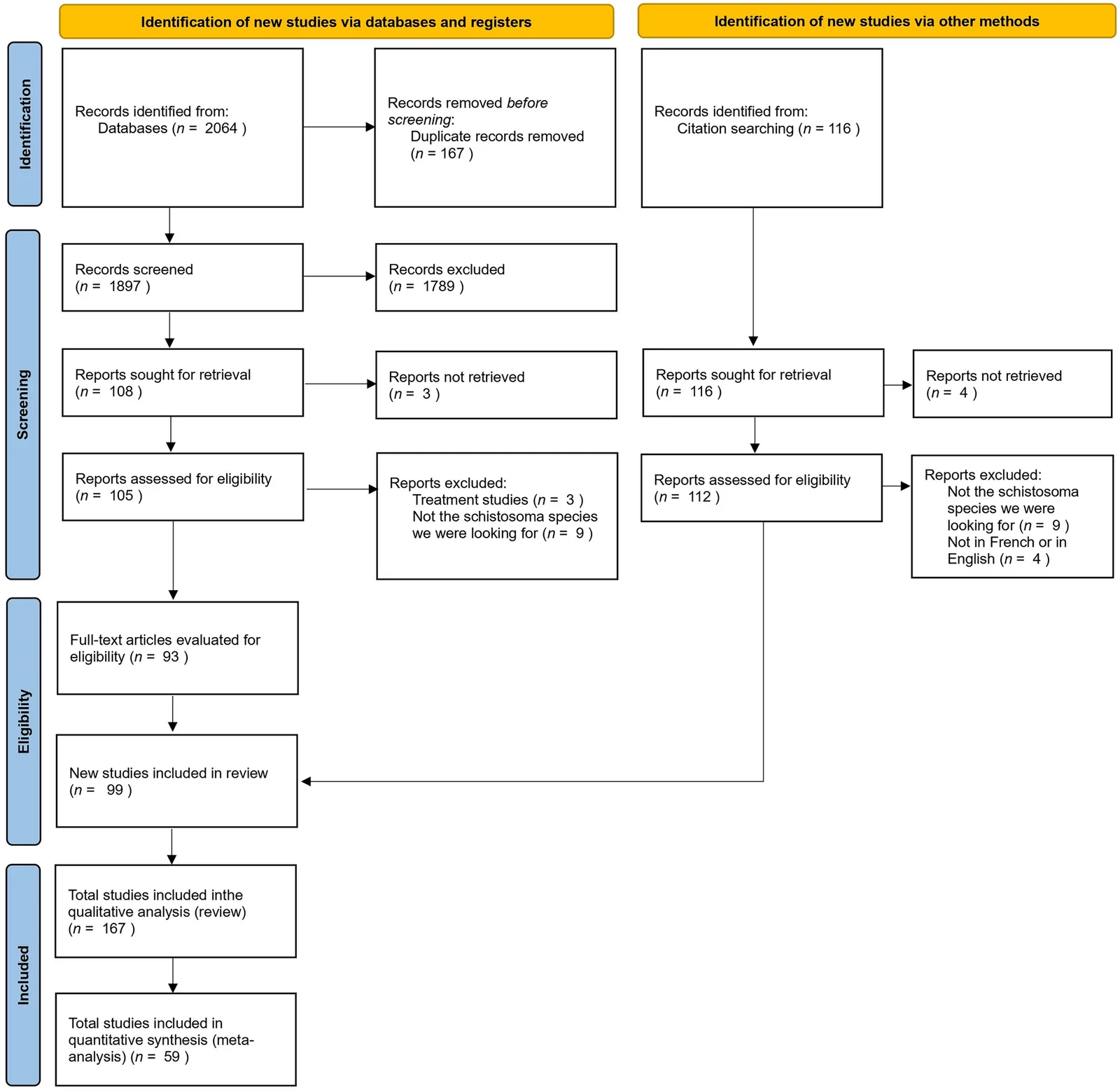
PRISMA 2020 flowchart of the process undertaken for the inclusion or exclusion of studies in the systematic review. PRISMA (Preferred Reporting Items for Systematic Reviews and Meta-Analyses) flowchart showing how 167 studies were obtained for the review. Of the 167 reports, 59 were included in the meta-analysis

Database searches identified 2,064 articles (Fig. 1). After removing duplicates, the titles and abstracts of 1,897 articles were reviewed, of which 105 articles were eligible for full review. A further 116 articles were identified by citation search, of which 112 were eligible. Of these 217 eligible articles, 25 were excluded for the following reasons (Fig. 1): 18 did not evaluate the *Schistosoma* species that we were including in this review, 3 focused on drug treatments and 4 were neither in French nor in English. A total of 192 articles were included in the systematic review to construct the qualitative part of this review, of which 59 were used for the quantitative synthesis (meta-analysis) only. Interestingly, 34 articles were split between quantitative and qualitative analysis. As a result, the qualitative analysis (systematic review) alone included 167 articles. The characteristics of the articles included in the meta-analysis are summarised in Appendix S3.

### Characteristics of the included studies

The characteristics of the 59 studies included in the meta-analysis are summarised in Appendix S3. All the studies were published between 1996 and 2023 and covered 14 countries—all on the African continent. Most studies were conducted in Nigeria (11/59, 18.64%). The selected studies included children and adults (both men and women). Co-infection between *S. haematobium* and *P. falciparum* were reported in 41 studies, 21 studies reported co-infection between *S. mansoni* and *P. falciparum*, 3 studies reported co-infection between *S. haematobium, S. mansoni* and *P. falciparum*, 1 study reported co-infection between *S. mansoni* and *P. vivax* and no study reported co-infection between *S. haematobium* and *P. vivax*.

### Risk of bias assessment

The methodological quality of the included studies was assessed using the Joanna Briggs Institute (JBI) critical appraisal checklist for studies reporting prevalence data. Overall, the majority of studies were classified as having a low risk of bias, and 7/59 studies were classified as having a moderate risk of bias, mainly due to limitations related to sampling strategies and variability in recruitment methods. Several studies also did not provide detailed information on response rates or justification for sample size. However, most studies used standardised and widely accepted diagnostic methods for the identification of *Plasmodium* and *Schistosoma* infections. The detailed assessment of the quality of each study is presented in Supplementary Table 1.

1. Epidemiology of co-infection

### Spatial distribution of *Plasmodium *and *Schistosoma*

The epidemiological data (59 articles in total) covered the prevalence of co-infection and the morbidity associated with dual infection. Helminthic and malarial infections spread when climatic and environmental conditions are favourable to their development, particularly in low-income communities that require the installation of essential elements such as drinking water, water sanitation or improved hygiene of premises [50, 51]. In Tanzania, studies suggests linking the control of malaria-schistosomiasis co-infection to types of agro-ecosystem after demonstrating that children in rice-irrigated ecosystems were more likely to be co-infected than in other measured ecosystems [9]. In countries such as Nigeria, Uganda, Ivory Coast and Ethiopia, helminth and malaria infections are highly prevalent and represent a persistent public health problem [52–55]. More than a third of the world's population, particularly those living in tropical and subtropical regions, are thought to have problems with concomitant infection by various species of *Plasmodium* and soil helminths [6].

**Fig. 2:**
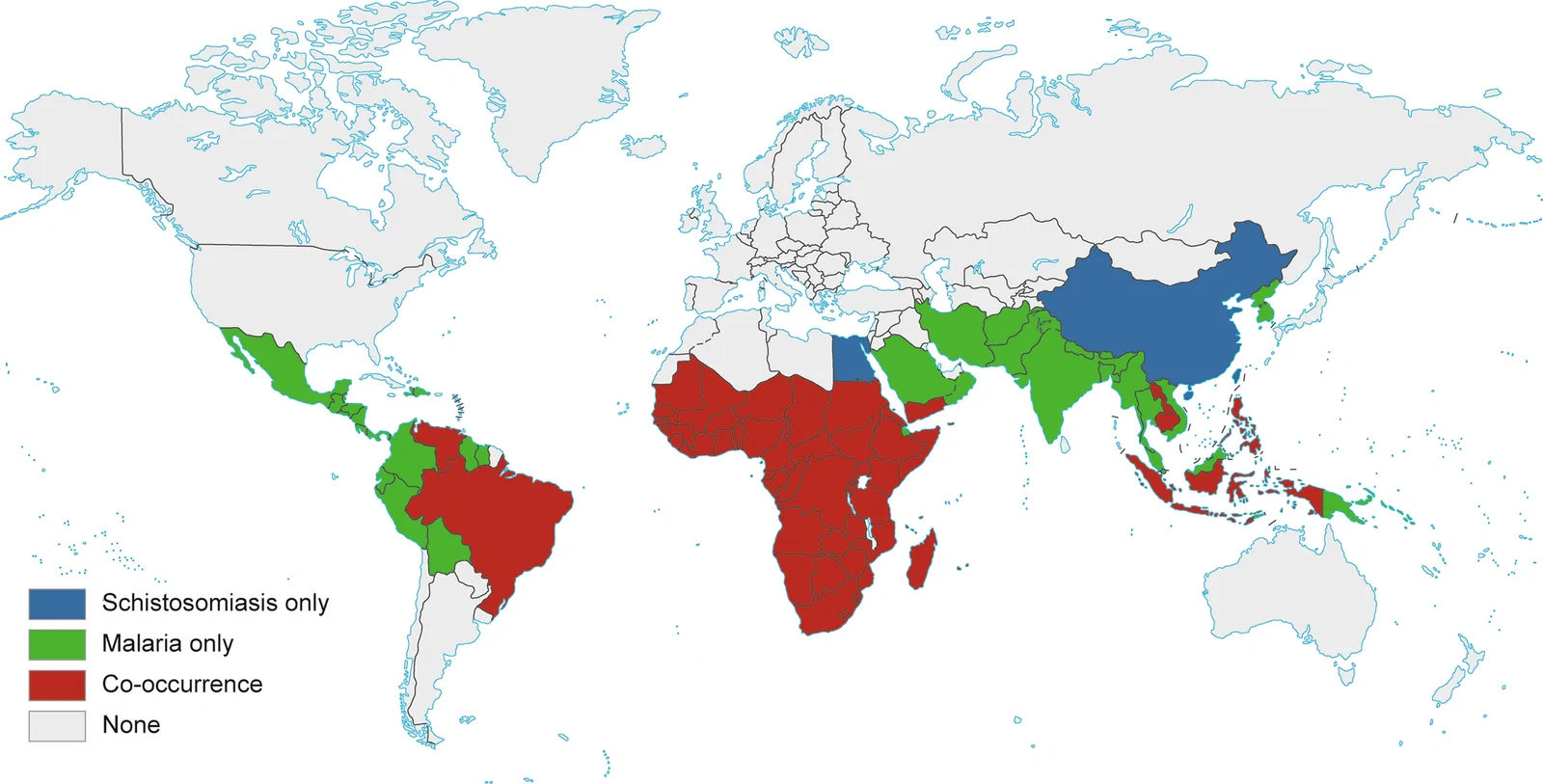
Global distribution of malaria, schistosomiasis, and their co-occurrence (2017–2022). Country shading indicates reported occurrence based on WHO Global Health Observatory data assessed in 2025: green—malaria only, blue—schistosomiasis only, red—co-occurrence of both infections, and grey—no reported occurrence. Map approval number: GS (2026) 4664

Both malaria and schistosomiasis are widespread in similar tropical and subtropical areas, particularly in sub-Saharan Africa (Fig. 2). Approximately 70% of the global burden of malaria is concentrated in sub-Saharan Africa [56] and 90% for schistosomiasis [57]. These co-infections are particularly well studied in Nigeria and Uganda, where 11 and 9 studies respectively have investigated the prevalence of *Plasmodium* and *Schistosoma* co-infection (Supp.Fig. 1). Similarly, the estimated prevalence of co-infection was recorded for Ethiopia, Ghana and Ivory Coast in 6 studies each, for Mali in 5 studies, for Tanzania, Senegal, Kenya and Gabon in 4 papers, for Cameroon in 3 papers, for Zimbabwe in 2 papers and for the Democratic Republic of Congo and Benin in 1 paper each (Supp.Fig. 1). Of the 59 articles considered for the epidemiological data, some analysed several countries. All the geographical areas listed in the studies analysed for this review are malaria- and schistosomiasis-endemic areas in sub-Saharan Africa.

### Descriptive prevalence of malaria and schistosomiasis single and co-infections

Malaria is currently naturally caused by 9 species of *Plasmodium*: *P. falciparum, P. vivax, P. malariae, P. ovale curtisi, P. ovale wallikeri* found only in humans, as well as *P. knowlesi, P. simium, P. brasilianum* and *P. cynomolgi* which, although mainly found in monkeys, is also a pathogenic agent in humans [58–60]. As far as schistosomiasis is concerned, there are currently more than 21 known species of *Schistosoma*, with 6 main species infecting Human (*S. haematobium*, *S. mansoni*, *S. intercalatum*, *S. japonicum*, *S. mekongi* and *S. malayensis*) [61, 62].

For this meta-analysis, we have chosen to focus exclusively on infections with *P. falciparum* and *P. vivax as well as S. mansoni* and *S. haematobium*—the most widespread representative species from both parasites in Africa [58, 62]. The prevalence of *P. vivax* infection remains poorly studied in Africa despite consistent evidence of the emergence of this species on the continent (Supp.Fig.  2A) [63, 64]. The results of the studies clearly show the research interest in the infection prevalence of the other three species, with a maximum of 106 analyses carried out on the infection prevalence for *P. falciparum* in the 59 studies, compared with 86, 41 and 6 prevalence recorded for *S. haematobium, S. mansoni* and *P. vivax* respectively. The prevalence rates assessed in the various studies included in the meta-analysis were also higher for *S. mansoni* and *P. falciparum* species, with peaks of 92% and 100% respectively. The maximum prevalence analysed for *S. haematobium* and *P. vivax* were 78% and 22% respectively. Furthermore, unlike the other three species, the lowest prevalence of *P. vivax* was 13% (0% was observed for other parasites). The average prevalence of *S. mansoni*, *S. haematobium*, *P. falciparum* and *P. vivax* in these studies was 21%, 30%, 40% and 17% respectively on the African continent (Supp.Fig. 2A).

In the present study, several types of co-infection were analysed, ranging from an association between two parasites to three. The most common and most studied co-infection was that between *P. falciparum* and *S. haematobium*, with 76 prevalence estimates recorded and an average prevalence of 17.3%. This was followed by co-infections between *P. falciparum* and *S. mansoni* with 30 recorded prevalence and an average of 9.1%, between *P. falciparum, S. haematobium* and *S. mansoni* with 6 recorded prevalence and an average of 4.6%, between *P. vivax* and *S. mansoni* with 1 census and 0.9% prevalence and between *P. vivax, P. falciparum* and *S. mansoni* with 1 census and 11.2% prevalence (Supp.Fig. 2B).

**Fig. 3:**
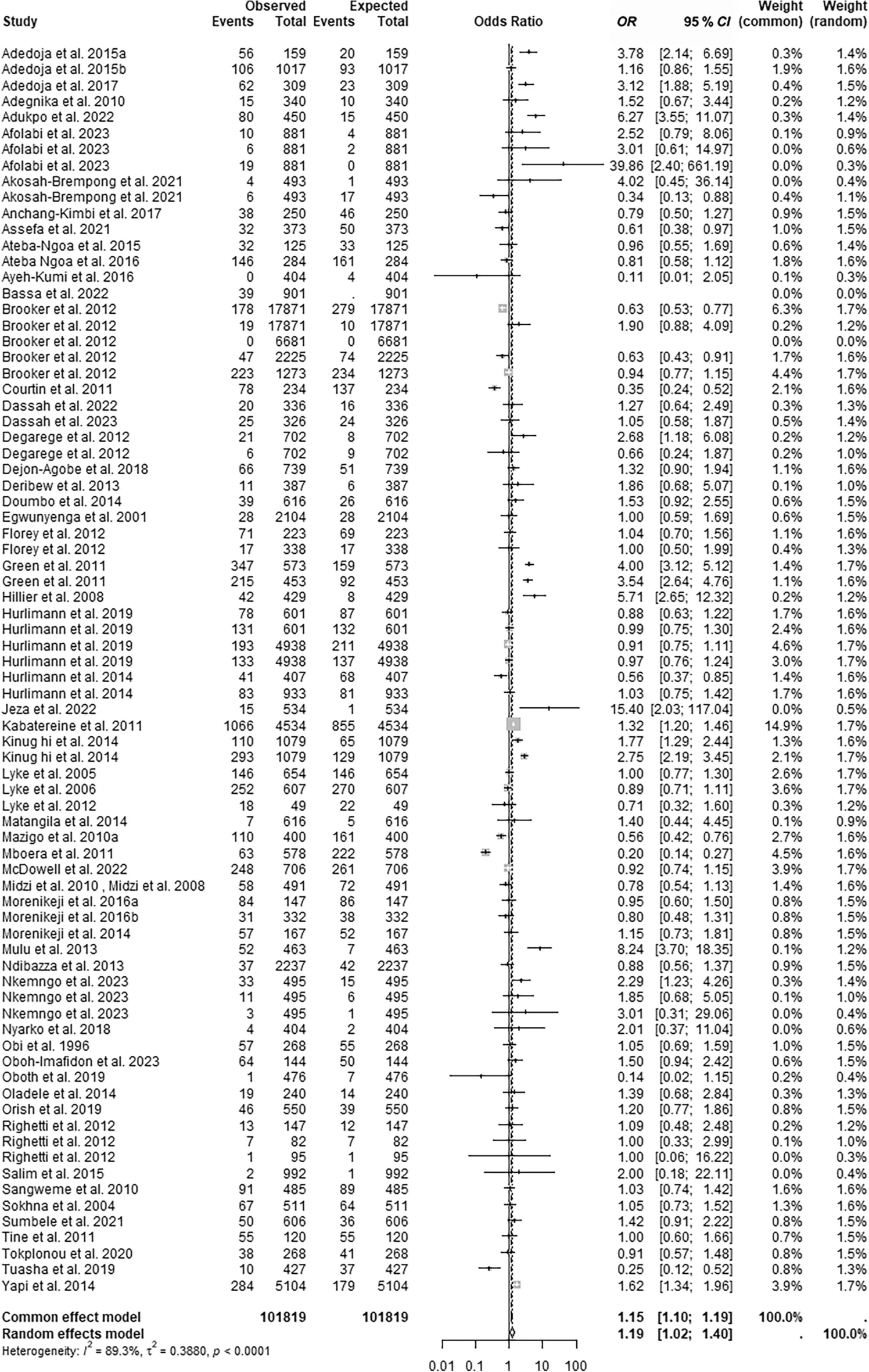
Forest diagram illustrating the results of the network meta-analysis for the overall prevalence of co-infections in studies that assessed the prevalence of malaria-schistosomiasis co-infections. Sensitive analysis comparing the difference between observed and expected co-infection. Blocks in the figure represent odds ratios, with error bars indicating 95% CI. Reports have been arranged alphabetically according to the first author. *OR* odds ratio, *CI* confidence interval, *I*^*2*^ heterogeneity coefficient, *t*^*2*^ inter-study variance

Differences in the prevalence of co-infections were found between the different African countries sampled in the meta-analysis studies. At the top of the list were Mali, with a prevalence of all co-infections combined of 36.6%, Nigeria with 25.8% and Gabon with 23.6%. Conversely, Benin, Ghana and Ethiopia had co-infections rates of less than 5% (Supp.Fig. 3). However, there has only been one study of the prevalence of co-infection in Benin.

### Meta-analysis of *Plasmodium* spp. and *Schistosoma* spp. co-infection over-dispersion

Meta-analysis of the prevalence of co-infection in all studies combined, in cohorts composed of men, women and children, revealed statistical heterogeneity (*I*^*2*^ = 89%; *P* -value < 0.0001) between studies (Fig. 3). Overall, the prevalence of co-infections appeared to be over-dispersed, with a random effect not including the 1 (95% *CI* 1.02, 1.40) (Fig. 3). The most overdispersed estimated prevalences were found in a cohort from Senegal with an odds ratio of 39.86 (95% *CI* 2.40, 661.19) and in second place in Kenya with an odds ratio of 15.40 (95% *CI* 2.03, 117.04). The highest estimated prevalence of co-infection was 74%, found in a very small population of 54 individuals, while the lowest estimated prevalence was 0%, found in two studies of 404 and 6681 persons (Supp. Table 2). Two studies by Bassa et al. 2022 and Lyke et al. 2012 did not report the number of mono-infected patients who developed malaria (Supp. Table 2). However, one of the studies may be subject to sampling bias since the patients selected were all positive for malaria (Supp. Table 2).

### Sex stratification reveals consistent over-dispersion

**Fig. 4:**
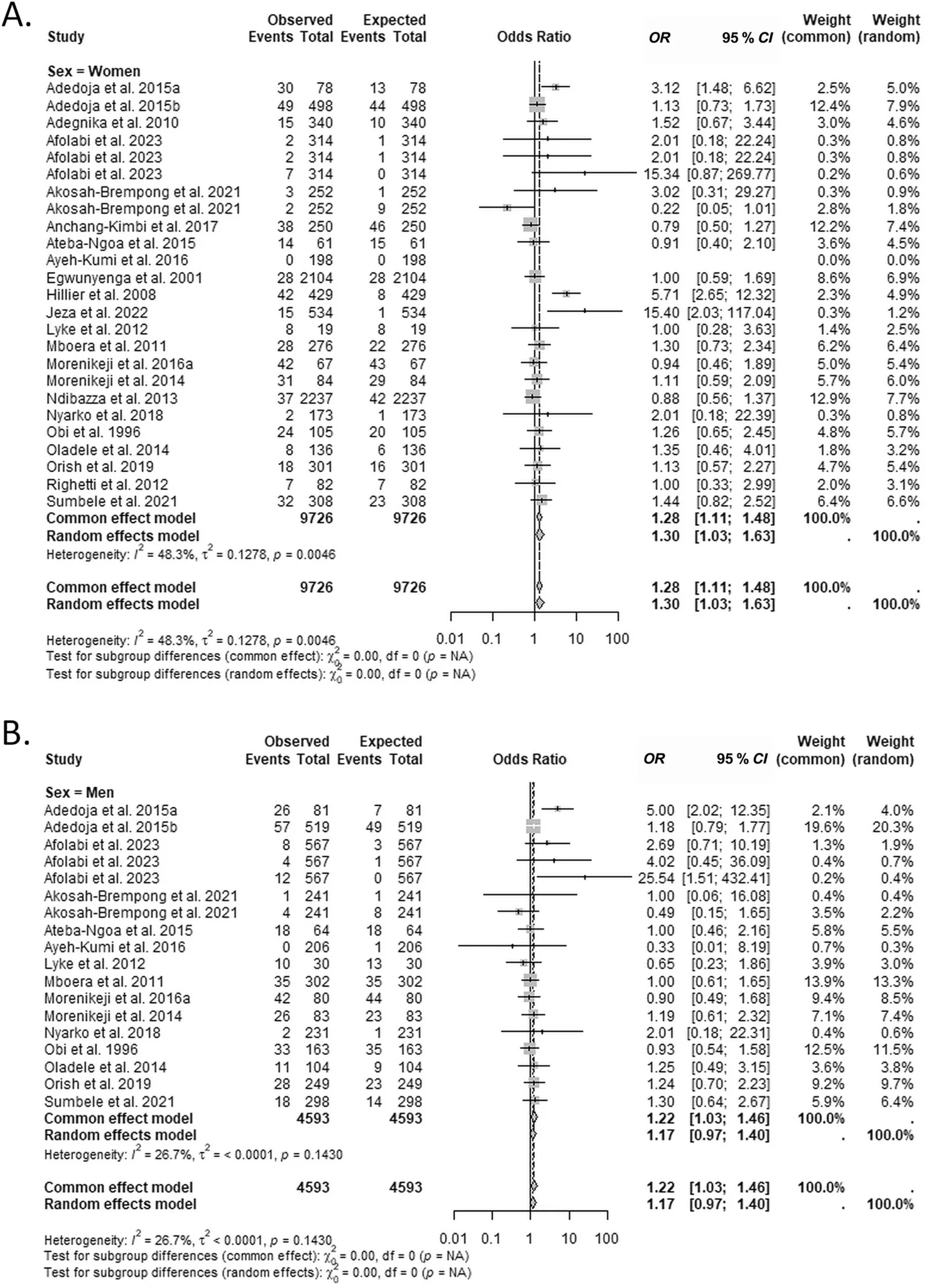
Forest diagram illustrating the results of the network meta-analysis for the overall prevalence of co-infections in women and men in studies that assessed the prevalence of malaria-schistosomiasis co-infections. Sensitive analysis comparing the difference between observed and expected co-infection in women and men. Blocks in the figure represent odds ratios, with error bars indicating 95% *CI*. Reports have been arranged alphabetically according to the first author. *OR* odds ratio, *CI* confidence interval, *I*^*2*^ heterogeneity coefficient,* t*^*2*^ inter-study variance

A total of 73,383 men and women were sampled for the prevalence of co-infection across the 59 studies listed. Some studies distinguished between men and women in their sampling cohorts, while others did not. A sub-group analysis was therefore carried out, with women and men on one side (Fig. 4). This highlighted the fact that, although a distinction is made between the two sexes in certain studies, there is no significant difference in the dispersion of the prevalence of co-infections between men and women (*x*^*2*^ = 0.15; *P* -value = 0.70). There was statistical heterogeneity (*I*^*2*^ = 48%; *P* -value = 0.0046), suggesting a moderate level of heterogeneity among the included studies for women, and statistical heterogeneity (*I*^*2 *^= 27%; *P* -value = 0. 1430), suggesting a low level of heterogeneity among the included studies for men, although the Egger's regression tests did not reach statistical significance (for women: Egger's test = 1.83, *P* -value = 0.0805; for men: Egger's test = 1.21, *P* -value = 0.2435). Overall, the meta-analysis by sex showed over-dispersion of co-infection in both 9726 women and 4593 men (Fig. 4) in 22 and 15 studies respectively. This corroborates the results of the overall meta-analysis (Fig. 3).

We may envisage that the distinction between men and women, in the cohorts, was decided on the basis of socio-economic status and behaviour, which differ between communities but also between the two sexes. Indeed, demography and climatic factors, social and behavioural factors are associated with the risk of infection by the two parasites [52]. Men are thought to be more exposed to the risk of schistosomiasis infection through long-term activities linked to water sources such as fishing or rice-growing [64, 65] or simply bathing or washing in natural water sources [67]. Moreover, in certain regions, religious and socio-cultural practices may limit women’s participation in activities such as swimming or fishing, potentially reducing their exposure to water-borne helminth infections [68, 69]. In contrast, patterns of exposure to malaria vectors appear to be highly context-dependent. In some settings, women may experience increased exposure to mosquito bites due to outdoor domestic activities conducted during peak vector biting hours, whereas in many other contexts, men show higher malaria prevalence, often associated with occupational or recreational outdoor activities [70–72]. Overall, sex-specific differences in malaria risk remain heterogeneous across settings and are influenced by a complex interplay of behavioral, environmental, and socio-cultural factors.

Co-infection rates of 46.8% were found among pregnant women in Cameroon, which can be explained mainly by the lack of water sources other than natural springs [73]. In contrast, co-infection prevalences of 23.8% and 20.8% were reported among pregnant women in Nigeria [74, 75]. This difference is mainly explained by the fact that pregnant women in some communities are denied access to natural water sources [74].

### General clinical and immunological features of malaria and schistosomiasis co-infection

A major consequence of co-infection between malaria and schistosomiasis is anaemia [30]. It is now known that these infections are both responsible for anaemia problems in humans, which could be exacerbated by the simultaneous presence of both parasites in the same host. For example, it has been shown that anaemia in Nigerien schoolchildren can be strongly influenced by a single infection with Plasmodium spp. or *S. haematobium*, but also by co-infection [10]. Other studies have also found higher anaemia in co-infected patients compared to *Plasmodium* infection [34, 41, 55, 73, 76–79] (Supp. Table 3). It has also been shown that anaemia is greater in cases of co-infection than in cases of *Schistosoma* infection [80]. However, other studies have reported contrasting results, showing that co-infection results in lower anemia levels compared to mono-infection with *Plasmodium* or *Schistosoma* [10, 46, 81–83] (Supp. Table 3). These discrepancies are hypothesized to arise from factors such as the genetic background of the human hosts and/or parasites, which may influence parasite-parasite and parasite-host interactions. In addition to the aforementioned data, co-infection with malaria and schistosomes is known to modify the immune profile of co-infected individuals, notably by altering the balance between the immune responses of TH1 response cells (helper T lymphocytes) and the TH2 response. Since *Schistosoma* infection induces a predominantly Th2-biased immune response during its chronic phase, whereas protozoan infections such as malaria are typically associated with Th1-type responses, co-infection can profoundly alter both the development of immune responses and the regulation of pre-existing immunity. This immune dysregulation may lead to reduced immunological control of *Plasmodium* infection. Evidence from both human and murine studies further indicates that effective resistance to either parasite relies on coordinated humoral and cell-mediated immune responses, and that malaria infection can significantly modulate antibody levels in vivo as well as cytokine responses to *Schistosoma mansoni* antigens in vitro [84]. Interestingly, *P. yoelii* infections reduced granuloma formation in the lungs of mice injected with *S. mansoni* eggs, indicating that malaria infection may influence granuloma formation in vivo [85].

Moreover, increasing evidence highlight the pathology seen in schistosomiasis and other parasitic diseases arises not only directly from parasite products but also from normal components of the immune response [86], in particular polymorphisms in cytokines such as IFN-γ, TNF-α, IL-4, IL-10, IL-13 and STAT-6 (Supp. Table 4). Some polymorphisms have been associated with susceptibility or resistance to *Schistosoma* infection [87]. While other cytokine polymorphisms may be affiliated with the activity, amount and timing of cytokine production, influencing malaria susceptibility and severity [88]. Given that Plasmodium spp. and Schistosoma spp. infections often coincide geographically in the same regions, it is crucial to know whether schistosomiasis infections modulate immune responses against the malaria parasite and affect its evolution.

2. Coinfection of species of Plasmodium and Schistosoma in experimental models

Several cases of co-infection with *Plasmodium* and *Schistosoma* in the murine model have already been tried, and numerous combinations have been tested: *S. mansoni* and *P. berghei* [85, 89–92], *S. mansoni* and *P. yoelii* [89, 93–95], *S. mansoni* and *P. chabaudi* [84, 89, 96] and *S. japonicum* and *P. berghei* [97, 98]. In addition, several of these studies have shown an effect of *Schistosoma* infection on the development of malaria, mainly reflected by an increase in parasitaemia [84, 94] as well as an increase in the duration of malaria infection, resulting in the death of the mice. Conversely, the study by Waknine-Grinberg et al. (2010) demonstrated that co-infection with *S. mansoni* and *P. berghei* in mice (not inbred) led to a change in *Plasmodium* infection and, in particular, increased survival in infected models. A change in cytokine expression due to the presence of *Schistosoma* was associated with a reduction in cerebral malaria.

However, co-infection with *Schistosoma* and *Plasmodium* is controversial in terms of its effects on either parasite. Indeed, some studies report a negative interaction between *Plasmodium* and *Schistosoma* [40, 48, 97–99] in which simultaneous infection with schistosomiasis and malaria resulted in lower densities of *Plasmodium* than in populations not infected with *Schistosoma,* suggesting that co-infection with schistosomiasis may have a protective effect against malaria [100]. Although some studies demonstrate the possibility of a negative association resulting from coinfection, other studies have demonstrated an additive or synergistic effect of coinfection [49, 101]. In an earlier study in animal models, co-infection with schistosomiasis prolonged the time to low-density malaria parasitaemia and induced anaemia compared with the group infected with malaria alone [102]. In many cases, however, it has been shown that previous infection with *Schistosoma* often has an effect on subsequent infection with a protozoan such as *Plasmodium* [100].

## Discussion

This systematic review and meta-analysis of 59 studies including 73,383 individuals across 14 malaria–schistosomiasis endemic African countries estimated an overall mean prevalence of co-infection of 13.41%, with substantial geographical variability ranging from 1.1% in Benin to 36.6% in Mali.Helminth–malaria co-infection has been increasingly investigated in recent years [18, 77, 103, 104], and our analysis, combining a co-endemicity map with a meta-analytical approach, highlights that co-infection between Plasmodium spp. and Schistosoma spp. is widespread across sub-Saharan Africa.

The overdispersion observed in *Plasmodium-Schistosoma* co-infections is probably explained by a complex set of biological, ecological and/or behavioural mechanisms. From an immunological and biological perspective, several hypotheses can be put forward. Co-infection may result from direct facilitation: a previous infection may alter the host's immune response and facilitate the establishment of a second parasite, for example through immunomodulation or inappropriate polarisation of the response [7, 30]. Similarly, the cumulative effect of physiological stress caused by a first infection can weaken the host (loss of resources, alteration of the immune barrier), increasing the likelihood that another parasite will establish itself [105–107]. Such mechanisms could explain why certain individuals infected with one parasite are more likely to acquire additional infections, contributing to the aggregated distribution of co-infections observed in endemic settings.

From an ecological point of view, the overdispersion of co-infection is largely influenced by the co-endemicity of the two parasites. The spatial overlap of parasite ecological niches is frequently invoked to explain co-infection patterns [8, 12]. Although this argument is more commonly used to justify coexistence rather than aggregation, repeated co-exposure driven by shared environmental conditions — such as the proximity of mosquito breeding sites and freshwater habitats harbouring intermediate snail hosts — may increase the probability of simultaneous exposure within specific subgroups of the population. Climatic factors, including rainfall, soil moisture and drought periods, further shape transmission dynamics by influencing the survival and development of parasite larval stages and determining seasonal transmission patterns [30]. In the context of climate change, increased environmental instability and shorter transmission windows may promote temporal overlap of parasite activity, potentially reinforcing co-infection clustering. Although malaria is often considered an acute infection compared to schistosomiasis, asymptomatic *Plasmodium* infections may persist for months or even years, particularly in endemic settings. In addition, malaria prevalence may fluctuate seasonally in many regions despite year-round transmission, which can influence reported co-infection rates and contribute to observed heterogeneity across studies.

Host-related factors also play a critical role in shaping co-infection overdispersion. Human behaviour strongly influences exposure risk, particularly among school-aged children, who are more likely to engage in outdoor activities and frequent contaminated water sources, thereby increasing their likelihood of acquiring multiple infections [12, 30]. Daily practices such as swimming, fishing, clothes washing and irrigated agriculture, as well as housing proximity to mosquito breeding sites or snail habitats, further contribute to heterogeneous exposure patterns. Beyond behavioural factors, intrinsic heterogeneity in host susceptibility has been described, whereby a small proportion of individuals — often referred to as “super-recipients” — exhibit increased susceptibility to multiple infectious agents due to genetic, physiological or immunological factors [104, 108, 109]. Such individuals disproportionately contribute to the burden of co-infection and are thought to play a major role in generating the observed patterns of overdispersion. [110, 111]. Gene–parasite interactions and environmental influences may further shape these susceptibility profiles [111, 112], while long-term co-adaptation mechanisms between parasites have also been suggested as a potential contributor to niche sharing and aggregated infection patterns [109, 110].

In short, the over-dispersion of *Plasmodium-Schistosoma* co-infection cannot be attributed to a single factor. It results from a complex interaction between biological mechanisms (immunomodulation, fragility, genetic susceptibility), ecological factors (niche overlap, climatic influence, vector ecology) and host-related factors (risky behaviours, super-receptors). Understanding these interactions is essential for better predicting the dynamics of co-infections and refining control strategies in co-endemic areas.

Among the populations analysed, pregnant women were frequently identified among individuals co-infected with *Plasmodium* and *Schistosoma*. It has been consistently shown that pregnant women and young children experience higher morbidity and mortality when infected with either malaria or schistosomiasis [73, 105, 112, 113], highlighting their vulnerability in co-endemic settings. Malaria-schistosomiasis co-infection is of particular concern in these groups, as it has been associated with more severe clinical manifestations and pathology compared to mono-infections [49, 101, 106], as well as with potential modulation of the host immune response resulting from interactions between the two parasites [107, 114].

School-aged children are a key population in studies of malaria-schistosomiasis co-infection, as they often exhibit higher infection intensities and an increased susceptibility compared to older individuals due to immature or developing immune responses [44, 115]. Early-life exposures, including potential in utero sensitisation, may further contribute to increased vulnerability. In addition, school-aged children frequently engage in high-risk behaviours such as bathing, swimming or washing in natural water bodies, which represent major transmission sites for schistosomiasis and may also increase exposure to malaria vectors. These environments may additionally include multiple contamination points, such as areas of open defecation, further increasing infection risk [115, 116]. Accordingly, high prevalences of malaria-schistosomiasis co-infection have been consistently reported among school-aged children in countries such as Nigeria, Uganda and Ghana [45, 51, 54, 76, 77, 117, 118]..

Experimental models have provided valuable insights into the pathological and immunological mechanisms underlying *Plasmodium*–-*Schistosoma* co-infection. In particular, murine models, which benefit from a well-characterised immune system and controlled experimental conditions, have been widely used to investigate parasite interactions, host immune modulation and disease outcomes. Although simian models have also been employed in a limited number of studies [99, 119], experimental mouse models have played a central role in elucidating the effects of co-infection on parasite virulence, host susceptibility and immune responses [120–122].

Despite the growing body of literature on malaria-schistosomiasis co-infection, systematic and comparable data on the impact of polyparasitism across age groups and diverse epidemiological and nutritional contexts remain limited. Furthermore, much of the morbidity associated with polyparasitism remains unclear and poorly understood, such as the non-health-related societal consequences of cognitive impairment caused by helminthic and malaria co-infections [123, 125]. Anaemia is also a burden associated with schistosomiasis and malaria, and various studies have shown that the prevalence of anaemia can be significantly increased in cases of co-infection [55, 77, 79].

Research carried out in various epidemiological contexts has shown that polyparasitism occurs at intervals that are variable and dissimilar to those expected in the hypotheses of independence [124–126]. Our meta-analysis confirmed an over-dispersion in the prevalence of co-infection compared with the expected results at the global level of the studies and between the sexes in the human host. There is increasing evidence that schistosomal infections are capable of altering susceptibility to clinical malaria [32, 36] and there are now studies focusing on the mechanisms of co-infection, although these remain rare [48, 82]. In animal models, it has been suggested that these co-infections have both synergistic and antagonistic effects [40, 84, 91, 94, 96]. Several hypotheses have therefore emerged to explain the interactions between *Schistosoma* and *Plasmodium*. Most studies on the interactions between helminths and malaria seem to indicate that helminth infection has a negative effect on the acquisition of immunity against malaria [81, 102, 127]. It has been proven on several occasions that helminthic infection modulates the immune system of their host with the aim of survival [107]. One hypothesis is that helminths promote the production of non-cytophilic antibodies via the cytokine milieu, resulting in individuals who are more susceptible to clinical malaria infections [30]. The presence of regulatory T cells intensifies during helminth infection, which can induce non-specific suppression by creating an anti-inflammatory environment [128]. This non-specific suppression could extend to the immune response of other pathogens such as *Plasmodium* [128]. Added to this is exposure to various environmental factors which, according to Smolen et al. 2014, could have an effect on the immune response of individuals. Indeed, this study compared the immune responses of children encountered on four different continents and demonstrated significant heterogeneity in innate cytokine responses in the different geographical areas sampled [112]. The differences in innate immune responses assessed were attributed to variations in environmental exposures such as feeding patterns, past infections, vaccination status, mode of delivery, region of residence, resource availability etc. [129].

It has also been shown that the genetics of individuals represent a factor that can lead not only to infectivity but also to the severity of the disease in patients exposed to the same rates of infection [130]. On the one hand, it seems that children born to mothers co-infected with *Schistosoma* and *Plasmodium* have a higher risk of malaria parasitaemia [131]. Some studies have also shown that a fetus that has been exposed to maternal infections can react with hyporeactivity of the T lymphocytes that subsequently induce a reduction in immunity to these two parasites [107, 132]. On the other hand, the study by Oboh-Imafidon et al. 2023 revealed that people carrying a heterozygous (CT) or mutant (TT) CD14 (Cluster of Differentiation 14) genetic variant were up to 58% more likely to be co-infected than to be infected by *Schistosoma* alone. CD14 is expressed by the majority of immune response cells—neutrophils, monocytes, macrophages—during the pro-inflammatory response, thus playing a key role in innate immunity and offering, in this case, protection against the severity of the disease relative to the egg load. Indeed, this study also revealed that *Schistosoma* egg counts were higher in individuals mono-infected with *Schistosoma* than in co-infected individuals carrying the mutant allele (TT) [114]. Although the results do not appear to be significant, other studies have shown a link between the number of eggs produced by schistosomes and the intensity of malaria infection during co-infection [31, 32, 48, 133]. Tokplonou et al. 2023 suggest that eggs may act to activate, suppress or regulate immune pathways.

Other, less obvious factors, such as pathogen species, microgeographical variations [30]or the age of individuals may also come into play to explain the high rates of co-infection found in the meta-analysis. Age, in particular, is a recurring factor, with children being more affected by polyparasitism in general [75, 134] and by schistosomiasis-malaria co-infection [41, 52, 103, 135].

The meta-analysis carried out between the sexes showed that there were no differences in co-infection rates between men and women, even though many studies report a different prevalence of infection between the two sexes for the two parasites [65–67, 70, 72].

### Limitations

This systematic review did not escape the limitations identified in the studies considered in the meta-analysis. Although the initial objective was to provide a global overview of malaria-schistosomiasis co-infection, the quantitative analysis was limited to studies conducted in Africa due to a lack of eligible data from other endemic regions, such as South America and parts of Asia. Consequently, the results cannot be fully generalised to all co-endemic contexts, where ecological, epidemiological and health system factors may differ. Most of the included studies were cross-sectional, which limited the ability to assess temporal dynamics of malaria–schistosomiasis co-infection. In addition, selection bias may have been introduced in studies that deliberately recruited infected or co-infected individuals, as well as through non-participation of eligible individuals in some settings. Seasonal variation in malaria transmission may also have influenced co-infection prevalence estimates. Heterogeneity across studies was further increased by differences in study design, population characteristics and implementation methodologies, and in some cases by limited sample sizes, which reduced statistical power to detect associations, including immunological effects.

With regard to malaria diagnosis, most studies relied on light microscopy, sometimes complemented by rapid diagnostic tests (RDTs). While microscopy remains a reference method for malaria diagnosis in endemic settings, it is known to have limited sensitivity for detecting low-density, asymptomatic and submicroscopic infections, as well as for identifying non-falciparum mono-infections. In addition, when RDTs were used, these primarily targeted *Plasmodium falciparum* antigens and may have failed to detect infections caused by other species such as P. vivax. Consequently, malaria prevalence — and therefore malaria–schistosomiasis co-infection estimates — may have been underestimated in some studies included in this review.

Similarly, no single diagnostic method provides absolute sensitivity for detecting either *Schistosoma* or *Plasmodium* infections. Although methods such as PCR for *Plasmodium* and Kato–Katz or urine filtration for *Schistosoma* are widely considered reference standards, they may still underestimate infection prevalence, particularly in cases of low-intensity or chronic infections.

Despite these limitations, this systematic review and meta-analysis provides an updated and comprehensive synthesis of the available evidence on malaria–schistosomiasis co-infection and contributes to a better understanding of the potential epidemiological interactions between these two parasites.

## Conclusions

This systematic review and meta-analysis demonstrates that the prevalence of malaria–schistosomiasis co-infection is higher than expected in endemic countries of sub-Saharan Africa, supporting our initial hypothesis of a non-independent distribution of these infections at the population level. The observed over-dispersion likely reflects a combination of shared ecological niches, heterogeneous host exposure and susceptibility, and potential biological interactions between parasites. Although the nature of the interactions between helminths, and more specifically between *Schistosoma* spp. and *Plasmodium* spp., remains poorly understood, ongoing research exploring parasite biology, genetic diversity, population dynamics, transmission patterns and molecular evolution is essential to elucidate the physiological and immune mechanisms underlying co-infection. Experimental studies using mouse or other animal models of co-infection under controlled conditions, combined with parasite genetics and genomics, may further clarify disease pathogenesis and host–parasite interactions. Beyond individual clinical management, these findings underscore the importance of considering polyparasitism in epidemiological surveillance and disease control strategies, supporting the need for integrated surveillance and coordinated interventions in co-endemic settings.

## Supplementary Information

Supplementary Material 1

Supplementary Material 2

Supplementary Material 3

Supplementary Material 4

Supplementary Material 5

Supplementary Material 6

Supplementary Material 7

Supplementary Material 8

Supplementary Material 9

## Data Availability

The data adopted in this meta-analysis are available from the corresponding author on reasonable request.

## Abbreviations

DALYs: Disability-adjusted life years
IC: Confidence indicator
JBI: Joanna Briggs institute
*OR*: Odds ratio
PCR: Polymerase chain reaction
PCT: Preventive chemotherapy and transmission
RDT: Rapid diagnostic test
