# Supplementary material for: Over-dispersion in malaria–schistosoma co-infection: insights from a meta-analytical approach and systematic review
Source: Infect Dis Poverty. 2026 Oct 9;15:107. doi: 10.1186/s40249-026-01490-1 (PMC13651093; doi:10.1186/s40249-026-01490-1)
Supplement: Supplementary file 1 [file 40249_2026_1490_MOESM5_ESM.docx]

**Supplementary Table 1 : Methodological quality assessment of the studies included in the meta-analysis using the Joanna Briggs Institute (JBI) Critical Appraisal Checklist for Studies Reporting Prevalence Data.**The risk of bias of each included study was evaluated across nine methodological domains: (1) appropriateness of the sample frame to address the target population, (2) appropriateness of participant recruitment methods, (3) adequacy of sample size, (4) detailed description of study subjects and setting, (5) sufficient coverage of the identified sample in the analysis, (6) validity of the methods used for the identification of the condition, (7) reliability and standardization of condition measurement across participants, (8) appropriateness of the statistical analysis, and (9) adequacy and management of the response rate. Each item was scored as Yes, No, Unclear, or Not applicable. An overall quality score was calculated as the proportion of “Yes” responses among applicable items. Based on this score, studies were classified as presenting low risk of bias (≥71% Yes responses), moderate risk of bias (50–70%), or high risk of bias (<50%).

| **Article** | **Was the sample frame appropriate to address the target population?** | **Were study participants sampled in an appropriate way?** | **Was the sample size adequate?** | **Were the study subjects and the setting described in detail?** | **Was the data analysis conducted with sufficient coverage**  **of the identified sample?** | **Were valid methods used for the identification of the**  **condition?** | **Was the condition measured in a standard, reliable way**  **for all participants?** | **Was there appropriate statistical analysis?** | **Was the response rate adequate, and if not, was the low**  **response rate managed appropriately?** | **Total Yes** | **Risk** |
| --- | --- | --- | --- | --- | --- | --- | --- | --- | --- | --- | --- |
| Adedoja et al. 2015a | YES | UNCLEAR | NO | YES | NOT APPLICABLE | YES | YES | YES | NOT APPLICABLE | 5/7 | **Moderate Risk** |
| Adedoja et al. 2015b | YES | YES | YES | YES | NOT APPLICABLE | YES | YES | YES | NOT APPLICABLE | 7/7 | **Low Risk** |
| Adedoja et al. 2017 | YES | YES | YES | YES | NOT APPLICABLE | YES | YES | YES | NOT APPLICABLE | 7/7 | **Low Risk** |
| Adegnika et al. 2010 | NO | NO | YES | YES | NOT APPLICABLE | YES | YES | YES | NOT APPLICABLE | 5/7 | **Moderate Risk** |
| Adukpo et al. 2022 | YES | YES | YES | YES | NOT APPLICABLE | YES | YES | YES | NOT APPLICABLE | 7/7 | **Low Risk** |
| Afolabi et al. 2023 | YES | YES | YES | YES | NOT APPLICABLE | YES | YES | YES | NOT APPLICABLE | 7/7 | **Low Risk** |
| Akosah-Brempong et al. 2021 | YES | YES | YES | YES | NOT APPLICABLE | YES | YES | YES | NOT APPLICABLE | 7/7 | **Low Risk** |
| Anchang-Kimbi et al. 2017 | NO | NO | NO | YES | NOT APPLICABLE | YES | YES | YES | NOT APPLICABLE | 4/7 | **Moderate Risk** |
| Assefa et al. 2021 | YES | YES | YES | YES | NOT APPLICABLE | YES | YES | YES | NOT APPLICABLE | 7/7 | **Low Risk** |
| Ateba-Ngoa et al. 2015 | YES | YES | NO | YES | NOT APPLICABLE | YES | YES | YES | NOT APPLICABLE | 6/7 | **Low Risk** |
| Ateba Ngoa et al. 2016 | YES | YES | NO | YES | NOT APPLICABLE | YES | YES | YES | NOT APPLICABLE | 6/7 | **Low Risk** |
| Ayeh-Kumi et al. 2016 | YES | YES | YES | YES | NOT APPLICABLE | YES | YES | YES | NOT APPLICABLE | 7/7 | **Low Risk** |
| Brooker et al. 2012 | YES | YES | YES | YES | NOT APPLICABLE | YES | YES | YES | NOT APPLICABLE | 7/7 | **Low Risk** |
| Courtin et al. 2011 | YES | YES | NO | YES | NOT APPLICABLE | YES | YES | YES | NOT APPLICABLE | 6/7 | **Low Risk** |
| Dassah et al. 2022 | YES | UNCLEAR | YES | YES | NOT APPLICABLE | YES | YES | YES | NOT APPLICABLE | 6/7 | **Low Risk** |
| Dassah et al. 2023 | YES | UNCLEAR | YES | YES | NOT APPLICABLE | YES | YES | YES | NOT APPLICABLE | 6/7 | **Low Risk** |
| Degarege et al. 2012 | YES | UNCLEAR | YES | YES | NOT APPLICABLE | YES | YES | YES | NOT APPLICABLE | 6/7 | **Low Risk** |
| Dejon-Agobe et al. 2018 | YES | YES | YES | YES | NOT APPLICABLE | YES | YES | YES | NOT APPLICABLE | 7/7 | **Low Risk** |
| Deribew et al. 2013 | YES | YES | YES | YES | NOT APPLICABLE | YES | YES | YES | NOT APPLICABLE | 7/7 | **Low Risk** |
| Doumbo et al. 2014 | YES | YES | YES | YES | NOT APPLICABLE | YES | YES | YES | NOT APPLICABLE | 7/7 | **Low Risk** |
| Egwunyenga et al. 2001 | NO | NO | YES | YES | NOT APPLICABLE | YES | YES | YES | NOT APPLICABLE | 5/7 | **Moderate Risk** |
| Florey et al. 2012 | YES | YES | YES | YES | NOT APPLICABLE | YES | YES | YES | NOT APPLICABLE | 7/7 | **Low Risk** |
| Green et al. 2011 | YES | YES | YES | YES | NOT APPLICABLE | YES | YES | YES | NOT APPLICABLE | 7/7 | **Low Risk** |
| Hillier et al. 2008 | NO | NO | YES | YES | NOT APPLICABLE | YES | YES | YES | NOT APPLICABLE | 5/7 | **Moderate Risk** |
| Hürlimann et al. 2019 | YES | YES | YES | YES | NOT APPLICABLE | YES | YES | YES | NOT APPLICABLE | 7/7 | **Low Risk** |
| Hürlimann et al. 2014 | YES | YES | YES | YES | NOT APPLICABLE | YES | YES | YES | NOT APPLICABLE | 7/7 | **Low Risk** |
| Jeza et al. 2022 | NO | UNCLEAR | YES | YES | NOT APPLICABLE | YES | YES | YES | NOT APPLICABLE | 5/7 | **Moderate Risk** |
| Kabatereine et al. 2011 | YES | YES | YES | YES | NOT APPLICABLE | YES | YES | YES | NOT APPLICABLE | 7/7 | **Low Risk** |
| Kinug’hi et al. 2014 | YES | YES | YES | YES | NOT APPLICABLE | YES | YES | YES | NOT APPLICABLE | 7/7 | **Low Risk** |
| Lyke et al. 2005 | YES | YES | YES | YES | NOT APPLICABLE | YES | YES | YES | NOT APPLICABLE | 7/7 | **Low Risk** |
| Lyke et al. 2006 | YES | YES | YES | YES | NOT APPLICABLE | YES | YES | YES | NOT APPLICABLE | 7/7 | **Low Risk** |
| Lyke et al. 2012 | YES | YES | NO | YES | NOT APPLICABLE | YES | YES | YES | NOT APPLICABLE | 6/7 | **Low Risk** |
| Lyke et al. 2012 | YES | YES | NO | YES | NOT APPLICABLE | YES | YES | YES | NOT APPLICABLE | 6/7 | **Low Risk** |
| Matangila et al. 2014 | YES | YES | YES | YES | NOT APPLICABLE | YES | YES | YES | NOT APPLICABLE | 7/7 | **Low Risk** |
| Mazigo et al. 2010a | YES | YES | YES | YES | NOT APPLICABLE | YES | YES | YES | NOT APPLICABLE | 7/7 | **Low Risk** |
| Mboera et al. 2011 | YES | YES | YES | YES | NOT APPLICABLE | YES | YES | YES | NOT APPLICABLE | 7/7 | **Low Risk** |
| McDowell et al. 2022 | YES | YES | YES | YES | NOT APPLICABLE | YES | YES | YES | NOT APPLICABLE | 7/7 | **Low Risk** |
| Midzi et al. 2010 & Midzi et al. 2008 | YES | YES | YES | YES | NOT APPLICABLE | YES | YES | YES | NOT APPLICABLE | 7/7 | **Low Risk** |
| Morenikeji et al. 2016a | YES | YES | NO | YES | NOT APPLICABLE | YES | YES | YES | NOT APPLICABLE | 6/7 | **Low Risk** |
| Morenikeji et al. 2016b | YES | YES | YES | YES | NOT APPLICABLE | YES | YES | YES | NOT APPLICABLE | 7/7 | **Low Risk** |
| Morenikeji et al. 2014 | YES | YES | NO | YES | NOT APPLICABLE | YES | YES | YES | NOT APPLICABLE | 6/7 | **Low Risk** |
| Mulu et al. 2013 | YES | NO | YES | YES | NOT APPLICABLE | YES | YES | YES | NOT APPLICABLE | 6/7 | **Low Risk** |
| Ndibazza et al. 2013 | NO | NO | YES | YES | NOT APPLICABLE | YES | YES | YES | NOT APPLICABLE | 5/7 | **Moderate Risk** |
| Nkemngo et al. 2023 | YES | YES | YES | YES | NOT APPLICABLE | YES | YES | YES | NOT APPLICABLE | 7/7 | **Low Risk** |
| Nyarko et al. 2018 | YES | YES | YES | YES | NOT APPLICABLE | YES | YES | YES | NOT APPLICABLE | 7/7 | **Low Risk** |
| Obi et al. 1996 | YES | YES | NO | YES | NOT APPLICABLE | YES | YES | YES | NOT APPLICABLE | 6/7 | **Low Risk** |
| Oboh-Imafidon et al. 2023 | YES | YES | NO | YES | NOT APPLICABLE | YES | YES | YES | NOT APPLICABLE | 6/7 | **Low Risk** |
| Oboth et al. 2019 | YES | YES | YES | YES | NOT APPLICABLE | YES | YES | YES | NOT APPLICABLE | 7/7 | **Low Risk** |
| Oladele et al. 2014 | YES | YES | NO | YES | NOT APPLICABLE | YES | YES | YES | NOT APPLICABLE | 6/7 | **Low Risk** |
| Orish et al. 2019 | YES | YES | YES | YES | NOT APPLICABLE | YES | YES | YES | NOT APPLICABLE | 7/7 | **Low Risk** |
| Righetti et al. 2012 | YES | YES | NO | YES | NOT APPLICABLE | YES | YES | YES | NOT APPLICABLE | 6/7 | **Low Risk** |
| Salim et al. 2015 | YES | YES | YES | YES | NOT APPLICABLE | YES | YES | YES | NOT APPLICABLE | 7/7 | **Low Risk** |
| Sangweme et al. 2010 | YES | YES | YES | YES | NOT APPLICABLE | YES | YES | YES | NOT APPLICABLE | 7/7 | **Low Risk** |
| Sokhna et al. 2004 | YES | UNCLEAR | YES | YES | NOT APPLICABLE | YES | YES | YES | NOT APPLICABLE | 6/7 | **Low Risk** |
| Sumbele et al. 2021 | YES | YES | YES | YES | NOT APPLICABLE | YES | YES | YES | NOT APPLICABLE | 7/7 | **Low Risk** |
| Tine et al. 2011 | YES | NO | YES | YES | NOT APPLICABLE | YES | YES | YES | NOT APPLICABLE | 6/7 | **Low Risk** |
| Tokplonou et al. 2020 | YES | YES | NO | YES | NOT APPLICABLE | YES | YES | YES | NOT APPLICABLE | 6/7 | **Low Risk** |
| Tuasha et al. 2019 | YES | UNCLEAR | YES | YES | NOT APPLICABLE | YES | YES | YES | NOT APPLICABLE | 6/7 | **Low Risk** |
| Yapi et al. 2014 | YES | YES | YES | YES | NOT APPLICABLE | YES | YES | YES | NOT APPLICABLE | 7/7 | **Low Risk** |
