# Supplementary material for: Over-dispersion in malaria–schistosoma co-infection: insights from a meta-analytical approach and systematic review
Source: Infect Dis Poverty. 2026 Oct 9;15:107. doi: 10.1186/s40249-026-01490-1 (PMC13651093; doi:10.1186/s40249-026-01490-1)
Supplement: Supplementary file 2 [file 40249_2026_1490_MOESM7_ESM.docx]

| **Article** | **Year of study** | **Geographic area** | **Sex** | **n=** | **Age** | **% S. mansoni** | **% S. haematobium** | **% P. falciparum** | **%P. vivax** | **%Coinfection** | **Anemia noted** | **Malnutrition** | **Limitations** |
| --- | --- | --- | --- | --- | --- | --- | --- | --- | --- | --- | --- | --- | --- |
| [Adedoja et al. 2015a](https://sci-hub.et-fine.com/10.4314/ijbcs.v9i1.13) | 2013 | Nigeria | M | 81 | 1-15 years old |  | 35,20% | 60,50% |  | 46,40% | NA | NA |  |
|  |  |  | W | 78 |  |  |  | 69,20% |  | 53,60% |  |  |  |
| [Adedoja et al. 2015b](https://journals.plos.org/plosntds/article?id=10.1371/journal.pntd.0003940) | 2012-2013 | Nigeria | M | 519 | 4-15 years old |  | 47,00% | 20,00% |  | 11,00% | Raised for plasmodium, schistosomiasis and coinfection | NA |  |
|  |  |  | W | 498 |  |  | 41,80% | 21,10% |  | 9,80% |  |  |  |
| [Adegnika et al. 2010](https://onlinelibrary.wiley.com/doi/full/10.1111/j.1365-3156.2010.02598.x) | 2003-2004 | Gabon | Pregnant W | 340 | <19->31 years old |  | 12,06% | 25,77% |  | 36,60% | NA | NA |  |
| [Afolabi et al. 2023](https://www.frontiersin.org/articles/10.3389/fpubh.2023.1087044/full) | 2021 | Senegal | M | 569 | 1-14 years old | 3,58% | 12,36% | 4,21% |  | P+SH = 1.41% P+SM = 0.7% P+S.M+SH= 2.29% | NA | 18.24% malnutrition within the group | The main limitation of our studies was the different timing of the implementation of the two studies, the first study being conducted in Saraya at the beginning of the malaria transmission season in June 2021, the second study being conducted at the end of malaria transmission in Diourbel. These time frames may have had an impact on our results. |
|  |  |  | W | 337 |  | 7,64% | 10,50% | 2,37% |  | P+SH = 0.58% P+SM = 0.58% P+S.M+SH= 2.04% |  |  |  |
| [Akosah-Brempong et al. 2021](https://www.ncbi.nlm.nih.gov/pmc/articles/PMC8680042/) | 2019 | Ghana | M | 241 | 2-15 years old | 23,10% | 1,60% | 14,10% |  | P+SH = 0.4% P+SM = 1.9% | NA | NA | An important limitation of the present study is the use of microscopy solely as a diagnostic method which could also have accounted for the relatively low parasite detection rate as it was possible to miss mild infections. |
|  |  |  | W | 252 |  | 18,20% | 1,70% | 20,20% |  | P+SH = 1.2% P+SM = 0.9% |  |  |  |
| [Al-Shehri et al. 2019](https://malariajournal.biomedcentral.com/articles/10.1186/s12936-019-2748-4) | 2015 | Uganda | M | 124 | 6.8 years old | NA |  | 63,90% |  | 44,50% | NA | NA | However, in Ebola risk areas, precautions should be taken in the collection and processing of stools. |
|  |  |  | W | 123 |  |  |  |  |  |  |  |  |  |
| [Al-Shehri et al. 2016](https://academic.oup.com/trstmh/article-abstract/110/10/597/2548938?redirectedFrom=fulltext&login=false) | 2015 | Uganda | M/W | 267 | 5-10 years old | 46,50% |  | 56,18% |  | 19,93% | 1/4 of the sampled children were anemic (25.7%) | NA |  |
|  |  |  |  |  |  |  |  |  |  |  |  |  |  |
| [Anchang-Kimbi et al. 2017](https://www.hindawi.com/journals/jpr/2017/6173465/) | 2014 | Cameroon | W pregnant | 250 | 14-40 years old |  | 46,80% | 39,20% |  | 15,20% | Mean hemoglobin level of 9g/dL. Anemia increased during coinfection (50 to 95.8%). Anemia prevalence of 88.8%. All coinfected cases were anemic and coinfection accounted for 93.8% of severe anemia. Coinfection contributes to the severity of anemia. Our study showed that the magnitude of S. haematobium egg count is significantly related to hemoglobin concentration confirming that urogenital schistosomiasis contributes to anemia | NA |  |
| [Assefa et al. 2021](https://www.ncbi.nlm.nih.gov/pmc/articles/PMC8669753/) | 2020 | Ethiopia | M | 193 | 5-35 years old | 50,80% |  | 23,60% |  | 8,60% | 25.9% (men) and 22.8% (women) anemic (anemia higher during coinfection) | 53.4% undernourished | First: the study is cross-sectional and cannot imply causal relationships; second: few people were infected with HBV, which may have limited the power and then the statistical significance; third: routine microscopy with a detection limit of 50–100 parasites/μL may seriously underestimate the reports of asymptomatic malaria infections, and the kato-taze method may underestimate the prevalence of intestinal parasites. |
|  |  |  | W | 180 |  | 57,20% |  |  |  |  |  | 46.1% undernourished |  |
| [Ateba-Ngoa et al. 2015](https://www.ncbi.nlm.nih.gov/pmc/articles/PMC4365807/) | 2011 | Gabon | M | 64 | 6-16 years old |  | 48,43% | 57,81% |  | 28,12% | Calculation of the hemoglobin level in the blood for each group. Mild anemia on average (11.76g/dl) | NA | An obvious limitation of the present study is that it is cross-sectional and it could be argued that it does not provide information on the history of past helminth infections that are capable of imprinting the disease on the host immune system. An additional limitation concerns the study sample size which may not be sufficient to detect an effect of helminths on P. falciparum modulated immune responses. |
|  |  |  | W | 61 |  |  | 52,46% | 47,54% |  | 22,95% |  |  |  |
| [Ayeh-Kumi et al. 2016](https://www.ncbi.nlm.nih.gov/pmc/articles/PMC4847346/) | 2012 | Ghana | M | 206 | 5-13 years old |  | 2,91% | 9,71% |  | 0,00% | General anemia of 73%.*Plasmodium* : 89% anemia*Schistosoma* : 50% anemia | 52.22% of subjects were malnourished. Malnutrition was the most powerful predictor of infection with*S. haematobium* |  |
|  |  |  | W | 198 |  |  | 2,02% | 8,59% |  |  |  |  |  |
| [Bassa et al. 2022](https://idpjournal.biomedcentral.com/articles/10.1186/s40249-021-00925-1/tables/1) | 2017 | Ivory Coast | M | 465 | 18-90 years old | 24,70% | 1,00% |  |  | P+SM = 18.7% | 20.5% anemic. No association found with infection with*S. mansoni* | 7.7% underweight |  |
|  |  |  | W | 436 |  | 21,60% |  |  |  |  |  |  |  |
| [Briand et al. 2005](https://pubmed.ncbi.nlm.nih.gov/15964953/) | 2001-2002 | Senegal | M | 284 | 3-15 years old |  | 69,72% | 58.25% (seasonal average) |  | NA | NA | NA | The tests were performed using thick blood smear and urine filtration techniques, which have high specificities but lower sensitivities. This lack of sensitivity is due to the limitations of the techniques, but also to fluctuations in the number of parasites in an individual's peripheral blood (for malaria) or urine (for schistosomiasis) over the course of a day. Our understanding of the mechanisms by which parasite interactions occur is still limited. |
|  |  |  | W | 239 |  |  | 57,74% | 41.75% (seasonal average) |  |  |  |  |  |
| [Brooker et al. 2012](https://www.ncbi.nlm.nih.gov/pmc/articles/PMC3274378/) | 2008-2009 | Kenya | M/W | 17871 | 10-13 years old | 0,90% | 24,40% | 6,40% |  | P+SH= 1% P+SM= 0.1% | NA | NA | Cross-sectional studies are inevitably subject to several limitations, including the potential for ecological bias. First, our estimates of Plasmodium and helminth infection relied on standard parasitological diagnosis. The ability of these techniques to detect infection is undoubtedly subject to error due to the insensitivity of light microscopy to detect malaria infection, daily and intraspecimen variation in fecal egg counts, and human error. Second, the current results represent a snapshot of the period 2006–2009, and it is conceivable that seasonality and intervention-related factors could confound results. |
|  |  | Ethiopia |  | 6681 | 9-12 years old | 2,80% | 0,00% | 0,10% |  | 0,00% |  |  |  |
|  | 2009 | Uganda |  | 2225 | 8-13 years old | 7,30% |  | 45,70% |  | 2,10% |  |  |  |
|  | 2006 | Uganda |  | 1273 | 7-13 years old | 32,90% |  | 55,80% |  | 17,50% |  |  |  |
| [Dassah et al. 2022](https://www.ncbi.nlm.nih.gov/pmc/articles/PMC9474855/) | 2018 | Ghana | M | 170 | 5-16 years old |  | 12,80% | 37,80% |  | 6,00% | *Plasmodium* : 17.8% anemia*Schistosoma*21.7% moderate anemia and 4.3% severe anemia Coinfection: 5% moderate anemia | NA | Furthermore, because malaria transmission is seasonal in Ghana, the potential for coinfection with the two parasites may differ considerably from one survey to another depending on the season of the school survey. Estimates of egg density from *S. haematobium* may have been negatively affected by the limited number of urine repeats processed in this study. Small sample size. |
|  |  |  | W | 166 |  |  |  |  |  |  |  |  |  |
| [Dassah et al. 2023](https://malariajournal.biomedcentral.com/articles/10.1186/s12936-023-04709-w) | 2022 | Ghana | M | 134 | 5-17 years old | 32,09% |  | 36,57% |  | 7,70% | General anemia of 40.5%.*Plasmodium* : 41.8%*Schistosoma*38.6% Coinfection: 64% | The high level of anemia in this study suggests poor nutrition and health in the region. Malnutrition was not assessed. | Main limitations; (i) the effect of other parasitic infections such as soil-transmitted helminths (STH) which were not assessed; (ii) non-infectious etiology of anemia such as malnutrition was not assessed; (iii) information on the cytokine profile of the infected participant was not assessed. Therefore, it was not possible to draw a firm conclusion on the correlation between concomitant malaria and Schistosoma infections and the risk of anemia; (iv) the scope of the present study is limited to schoolchildren aged 5–17 years and may not be sufficient for planning community interventions; (v) Although malaria microscopy and Kato Katz techniques are the gold standards for malaria and S. mansoni diagnosis respectively, PCR technology could increase the detection sensitivity because all participants were asymptomatic and the parasite load could be below the microscopy threshold, but PCR was not used due to resource constraints. |
|  |  |  | W | 192 |  | 20,31% |  | 24,47% |  |  |  |  |  |
| [Degarege et al. 2012](https://www.ncbi.nlm.nih.gov/pmc/articles/PMC3519704/) | 2010-2011 | Ethiopia | M | 522 | 1-82 years old | 13,60% |  | 13,60% | 15,80% | P.F+S= 23.1% P.V+S= 7.7% | Higher prevalence of anemia in coinfection | NA | The cross-sectional nature of the study design was a limitation in drawing conclusions about malaria risk or incidence due to prior helminthic infection status. |
|  |  |  | W | 543 |  | 9,70% |  | 12,30% | 13,00% |  |  |  |  |
| [Dejon-Agobe et al. 2018](https://journals.plos.org/plosntds/article?id=10.1371/journal.pntd.0006663) | 2012-2014 | Gabon | M | 388 | 6-16 years old |  | 30,90% | 24,70% |  | 8,93% | NA | NA |  |
|  |  |  | W | 351 |  |  | 29,60% | 20,20% |  |  |  |  |  |
| [Deribew et al. 2013](https://www.ncbi.nlm.nih.gov/pmc/articles/PMC3634929/) | 2013 | Ethiopia | M | 216 | 5-15 years old |  | 24,54% | 6,20% |  | 2,84% | There was a higher percentage of anemic patients (81.81%) in co-infected cases than in malaria (33.3%) or schistosomiasis (38.94%) cases. Hemoglobin level was negatively correlated with the number of S. haematobium eggs/10 mL of urine (r=-0.6) and malaria parasitemia (r=-0.53). | NA |  |
|  |  |  | W | 171 |  |  |  |  |  |  |  |  |  |
| [Doumbo et al. 2014](https://www.ncbi.nlm.nih.gov/pmc/articles/PMC4161351/) | 2011-2012 | Mali | M | 323 | 3 months - 25 years old | 0,16% | 11,14% | 47,57% |  | 6,33% | We found a significant interaction between anemia and infection with*S. haematobium* , the protective effect of*S. haematobium* being apparent only in individuals without anemia. More severe egg burden has been associated with increased hematuria and anemia | NA | An important limitation of our study is that we were unable to repeat urogenital schistosomiasis surveillance to determine reinfection frequencies in our cohort. Finally, unknown genetic, behavioral, or environmental factors that specifically co-associate with co-infected individuals and reduce malaria risk may confound the results of this study. |
|  |  |  | W | 293 |  |  |  |  |  |  |  |  |  |
| [Egwunyenga et al. 2001](https://www.scielo.br/j/mioc/a/FyjTg5vvDFnj4RTRc7bZ4Xv/?lang=en) | 1997-1998 | Nigeria | W pregnant | 2104 | NA | 3,40% |  | 36,20% |  | 7,16% | Generalized anemia. More significant in coinfection | NA |  |
| [Fairley et al. 2013](https://www.ncbi.nlm.nih.gov/pmc/articles/PMC3541745/) | 2000-2005 | Kenya | W pregnant | 696 | <20->30 |  | 30,60% | 42,70% |  | 29.5% helminth coinfection | NA | NA | We did not include other measures of adverse birth outcomes, such as preterm delivery or stillbirth. Assessing these outcomes may have revealed more effects of helminth infection or coinfection on birth weight. Furthermore, infection was only tested at the time of delivery, and given the high prevalence of infection, it is likely that some of the uninfected women were, in fact, infected at some point during their pregnancy. Other maternal factors not included in this study that may have been confounders include maternal height and smoking status. |
| [Florey et al. 2012](https://www.ncbi.nlm.nih.gov/pmc/articles/PMC3404100/) | 2006 | Kenya | M | 112 | 8-17 years old |  | 40,80% | 75,30% |  | 31,80% | NA | NA | Our cross-sectional design may have limited our estimation of the risk of continuous infection, particularly for malaria, given annual fluctuations in exposure related to weather conditions. |
|  |  |  | W | 111 |  |  |  |  |  |  |  |  |  |
|  |  |  | M | 105 | 18 years old + |  | 14,50% | 34,00% |  | 5,00% |  |  |  |
|  |  |  | W | 233 |  |  |  |  |  |  |  |  |  |
| [Getie et al. 2015](https://bmcresnotes.biomedcentral.com/articles/10.1186/s13104-015-1468-2) | 2013 | Ethiopia | M | 130 | 6->30 years old | NA |  | 71,70% | 25,90% | 21.5% coinfection (PF+PV+SH) in men, 16% (PF+PV+SH) in women. PF+SH= 15.6% PV+SH= 3.9% | More severe anemia when coinfected (35%) than in those infected only with malaria (25.5%) | NA |  |
|  |  |  | W | 75 |  |  |  |  |  |  |  |  |  |
| [Green et al. 2011](https://sci-hub.hkvisa.net/10.1017/s0031182011001016) | 2009 | Uganda - Lake Albert | M/W | 573 | 0.4-6 years old | 45,90% |  | 60,60% |  | 28,70% | average of 101.0g/L, prevalence of 68.9% | NA |  |
|  |  | Uganda - Lake Victoria |  | 453 | 2-6 years old | 40,70% |  | 47,50% |  | 20,10% | average of 116.8g/L, prevalence of 27.3% |  |  |
| [Hillier et al. 2008](https://www.ncbi.nlm.nih.gov/pmc/articles/PMC2886962/) | 2003-2005 | Uganda | W pregnant | 2507 | 14-47 years old | 18,00% |  | 11,00% |  | 9,80% | Only non-anemic women were accepted into the study. | NA |  |
| [Hürlimann et al. 2019](https://www.ncbi.nlm.nih.gov/pmc/articles/PMC6343929/) | 2011-2013 | Ivory Coast - community | M/W | 601 | 5-18 years old | 26,10% | 17,10% | 84,40% |  | P+SH= 73.87% P+SM= 82.86% | 40.4% anemia | 26% malnutrition | The diagnostic approach, especially for the detection of helminth infections, lacks sensitivity and, therefore, infection rates may be underestimated. Regarding anemia and splenomegaly, especially the former is multifactorial and can be caused by hematological diseases, inflammation and nutritional deficiencies, in addition to helminth infection and malaria. |
|  |  |  |  | 706 | 38.7 years old | 20,80% | 4,80% | 45,60% |  | NA | 28.3% anemia | 4.7% malnutrition |  |
|  |  | Ivory Coast - national |  | 4938 | 5-18 years old | 3,70% | 5,70% | 75,00% |  | P+S/H= 68.81% P+SM= 36.77% | 28.8% anemia | 28.4% malnutrition |  |
| [Hürlimann et al. 2014](https://www.ncbi.nlm.nih.gov/pmc/articles/PMC3942297/) | 2011 | Ivory Coast - Sahoua | M/W | 526 | <5->40 years old | 1,90% | 25,90% | 59,70% |  | P+SM= 8.9% P+SH= 10% | Strong positive association of anemia with *P. falciparum.*Overall prevalence of 19.5%. Anemia did not differ between boys and girls, but was associated with female sex in adulthood (age >18 years). | Malnutrition associated with both schistosome species. More common in boys than in girls (42.4% vs. 28.7%). 19% of all study participants showed signs of moderate or severe malnutrition. |  |
|  |  | Ivory Coast - Old crossroads |  | 407 |  | 28,40% |  | 70,10% |  |  |  |  |  |
| [Jeza et al. 2022](https://bmcpublichealth.biomedcentral.com/articles/10.1186/s12889-022-12526-0) | 2018 | Kenya | W | 534 | 15-50 years old |  | 3,74% | 4,87% |  | Pregnant women: 2.6% Non-pregnant women: 0.2% | NA | NA | The sensitivity of the Kato Katz STH diagnostic method may not select a low prevalence and intensity in an endemic area. A much more sensitive method is therefore recommended for future studies on the prevalence and intensity of schistosomiasis and STH in the study area. Second, we used a single-day urine examination which may have led to missing some positive cases of Hematobium given the low prevalence and intensity observed in the study area. In addition, we needed a much larger sample size to make a more reasonable comparison of the different results. |
| [Kabatereine et al. 2011](https://www.ncbi.nlm.nih.gov/pmc/articles/PMC3270004/) | 2009-2010 | Uganda | M/W | 4534 | 10-14 years old | 40,80% |  | 46,44% |  | 23,51% | NA | NA |  |
| [Kinug'hi et al. 2014](https://www.ncbi.nlm.nih.gov/pmc/articles/PMC3906044/) | 2006 | Tanzania | M | 759 | 3-13 years old | 39,70% | 19,70% | 29,80% |  | P.F+SM = 27.2% P.F+SH = 10.2% P.F+S.M+SH = 7% | The prevalence of anemia was 34.4% and was significantly associated with malaria, S. haematobium infection and multiple parasitic infections. The highest prevalence of anemia (60%) was in those co-infected with 3 parasites: P. falciparum, S. mansoni and Haematobium. 1% of children were severely anemic. | NA | Lack of information on household, socioeconomic status, and environmental factors that have been shown to influence the occurrence of coinfections by other studies. Lack of information on other causes of anemia such as malnutrition was another limitation. |
|  |  |  | W | 787 |  |  |  |  |  |  |  |  |  |
| [Le Hesran et al. 2004](https://sci-hub.yncjkj.com/10.1016/j.trstmh.2003.10.009) | 2001-2002 | Senegal | M/W | 105 | 6.6 years old |  | 5,00% | 100% (only malaria cases studied) |  | 8,10% | NA | NA |  |
| [Lemaitre et al. 2014](https://www.ncbi.nlm.nih.gov/pmc/articles/PMC3919243/) | 2002-2003 | Senegal | M | 108 | 5-13 years old |  | 40,45% | NA |  | NA | NA | NA |  |
|  |  |  | W | 70 |  |  | 23,59% |  |  |  |  |  |  |
| [Lyke et al. 2005](https://www.ncbi.nlm.nih.gov/pmc/articles/PMC2738948/) | 2002-2003 | Mali | M | 322 | 4-14 years old |  | 50,00% | 83,20% |  | 41,51% | No anemia found between healthy and infected groups | NA |  |
|  |  |  | W | 332 |  |  | 50,30% |  |  |  |  |  |  |
| [Magalhaes et al. 2011](https://www.ncbi.nlm.nih.gov/pmc/articles/PMC3110251/) | 2003-2006 | Burkina Faso, Ghana and Mali | M | 3670 | 1-4 years old |  | 26,80% | 52,00% |  | NA | In the four countries, the prevalence of mild, moderate, and severe anemia was 21%, 66%, and 13% in Burkina Faso; 28%, 65%, and 7% in Ghana; and 26%, 62%, and 12% in Mali. Risk of anemia attributable to*P. falciparum* : 36.8%; to*S. heamatobium* : 14.9% | Prevalence of underweight at 71.2%. Severe malnutrition is responsible for approximately 2.5 million cases of anemia in these regions. |  |
|  |  |  | W | 3477 |  |  |  |  |  |  |  |  |  |
| [Matangila et al. 2014](https://www.ncbi.nlm.nih.gov/pmc/articles/PMC4220949/) | 2012 | DR Congo | M | 318 | 4-13 years old | 4,71% |  | 18,51% |  | 1,14% | Prevalence of anemia at 41.6%, strongly linked to infection*Plasmodium* And*Schistosoma* | Some cases of growth retardation, thinness and underweight | First, given the high refusal rate (32.8%), which may lead to selection bias, the reported data may not be representative of the schools surveyed. Second, conventional microscopy, the laboratory method used in this study, is not sensitive enough to detect low-grade asymptomatic infections. Therefore, a highly sensitive PCR-based diagnosis, which is between 2.7 and 8.6 times more sensitive than conventional microscopy in detecting malaria parasites in apparently healthy children, would provide a more accurate picture of asymptomatic cases of plasmodium. |
|  |  |  | W | 298 |  |  |  |  |  |  |  |  |  |
| [Mazigo et al. 2010](https://pubmed.ncbi.nlm.nih.gov/24409638/) | 2009 | Tanzania | M/W | 400 | 8-16 years old | 42,25% |  | 14,00% |  | 27,50% | NA | NA |  |
| [Mboera et al. 2011](https://www.sciencedirect.com/science/article/pii/S0001706X11001902) | 2005 | Tanzania | M | 302 | 8.07 years old |  | 30,79% | 37,75% |  | 11,59% | General anemia 61.94%. Severe anemia (Hb<8) 10.21%. There was no significant difference in the prevalence of anemia between simple infections and co-infections ( p > 0.05). | NA | As the study was cross-sectional in nature, the temporal association between co-infections could not be determined. It is therefore important that longitudinal studies are undertaken to identify clear associations between co-infections in space and time. |
|  |  |  | W | 276 | 7.84 years old |  | 23,91% | 32,61% |  | 10,14% |  |  |  |
| [McDowell et al. 2022](https://www.ncbi.nlm.nih.gov/pmc/articles/PMC9200150/) | 2009-2011 | Uganda | M | 372 | <1-5 years old | 52,70% |  | 73,65% |  | 35,20% | NA | NA |  |
|  |  |  | W | 334 |  | 47,30% |  |  |  |  |  |  |  |
| [Midzi et al. 2010 & Midzi et al. 2008](https://www.sciencedirect.com/science/article/pii/S0001706X10000392) | 2004 | Zimbabwe | M | 307 | 5-15 years old | 22,70% | 52,30% | 27,90% |  | S.M+S.H+P= 11.8% P+SH= 60.7% P+SM= 25.8% | Anemia was more significant in co-infections than in mono-infections, particularly for plasmodium and schistosoma. The prevalence of anemia was 48.4%. Males had a significantly higher mean Hb level than females. | NA | The limitation of our study is that it is cross-sectional, so inference about the causality of polyparasitism on anemia should be taken with caution because the design makes it impossible to retrospectively determine whether polyparasitism occurs or follows anemia. Causality can be better determined by prospective studies. Another limitation of our methodology could be the interpretation of the Kato Katz results based on the examination of a single Kato Katz thick smear prepared per stool sample per day for two successive days instead of the more acceptable double thick smears prepared per sample per day on at least two different days |
|  |  |  | W | 302 |  |  |  |  |  |  |  |  |  |
| [Morenikeji et al. 2016](https://www.sciencedirect.com/science/article/pii/S1876034115001409?via%3Dihub) | NA | Nigeria | M | 94 | 6-18 years old |  | 74,47% | 73,75% |  | 52,50% | NA | NA | A limitation of this study is the use of a single urine and blood sample for the diagnosis of schistosomiasis and malaria. This could lead to underreporting of the true infection status. Furthermore, no studies on the bionomics of the intermediate hosts of snails and mosquito vectors were conducted to further support the effects of topography on the distribution of schistosomiasis and malaria in the region. |
|  |  |  | W | 79 |  |  | 75,95% | 83,58% |  | 62,69% |  |  |  |
| [Morenikeji et al. 2014](https://www.sciencedirect.com/science/article/pii/S2212832814001234?via%3Dihub) | 2013 | Nigeria | M | 101 | 6-18 years old |  | 47,52% | 59,04% |  | 31,32% | The risk of developing low hemoglobin concentration was higher in children with P. falciparum infection. The risk associated with low hemoglobin in single S. haematobium infection was increased in coinfection with P. falciparum. However, schistosomiasis did not appear to be the cause of the anemic state in children infected with S. haematobium in the present study. |  | The high level of co-infection of the two parasites suggests some overlap in their epidemiology, including**lack of awareness and provision of basic social amenities** could be the factors involved. Limited resources/low level of target subject population |
|  |  |  | W | 101 |  |  | 56,43% | 60,71% |  | 36,90% |  |  |  |
| [Muhangi et al. 2007](https://www.ncbi.nlm.nih.gov/pmc/articles/PMC1950430/) | 2003-2005 | Uganda | W pregnant | 2507 | 14-47 years old | 18,30% |  | 10,90% |  | NA | The prevalence of anemia was 39.7%. Anemia showed little association with the presence of helminths, but showed a strong association with malaria. The prevalence of anemia decreased with age. The prevalence of anemia was higher in women with heavy S. mansoni infection than in those with lesser or no infection, but the number of these women was small and the effect was not statistically significant. | NA | Our research on intestinal helminths used only one stool sample from each woman, meaning that a proportion of women with low-level infections will have been misclassified as uninfected and that estimates of intensity will have been imprecise (unlikely for*S. mansoni*). |
| [Mulu et al. 2013](https://malariajournal.biomedcentral.com/articles/10.1186/1475-2875-12-227) | 2006 | Ethiopia | M | 190 | <9-50+ years old | 32,63% |  | 44,21% | 28,95% | S.M+P.F+PV = 22.61% | NA |  | It is difficult to draw definitive conclusions about the epidemiological interaction of helminths and malaria infection from such a cross-sectional study. The effect of specific helminths on malaria severity and the mechanisms leading to it need to be determined in longitudinal studies involving a larger number of clinical malaria cases. |
|  |  |  | W | 162 |  | 23,46% |  | 40,12% | 25,31% |  |  |  |  |
| [Ndibazza et al. 2013](https://academic.oup.com/jid/article/208/12/2007/2192713) | 2003-2005 | Uganda | W pregnant | 2289 | NA | 18,13% |  | 10,31% |  | 1,65% | Malaria during pregnancy has been associated with an increased risk of maternal anemia. But there is little association between maternal helminths and maternal anemia. | NA | Our main limitation was the use of single samples for verification of maternal malaria and helminth infections. Although microscopy is the gold standard for malaria diagnosis, sensitivity is low during pregnancy due to low parasite density and placental sequestration. Misclassification of malaria-infected mothers classified as seronegative would weaken the strength of the observed associations between maternal and infant malaria. Similarly, for helminths, some mothers may have been misclassified as uninfected. Underestimation of these key exposures may also explain the lack of interaction between maternal malaria and helminths. Finally, we cannot exclude the possibility that confounding by unmeasured covariates may explain some of the observations. |
| [Nkemngo et al. 2023](https://www.ncbi.nlm.nih.gov/pmc/articles/PMC10389741/) | 2021 | Cameroon | M | 165 | 3-9 years old | 5,16% | 12,26% | 47,10% |  | P+SH= 8.69% P+SM= 3.64% P+S.H+SM= 0.61% | Prevalence of anemia at 32.9% categorically associated with*P. falciparum* (45.8%), and to coinfections P.F+SH (11.5%) and P.F+SM (3.9%) | NA | The cross-sectional design of this study is a limitation. Similarly, this study focused on quantifying the prevalence of schistosomiasis primarily in school-aged children without including other high-risk groups, including pregnant women and occupation-related residents. Furthermore, the diagnosis of schistosomiasis was based on parasitological capture using a single collection of urine and stool samples instead of the standard three consecutive samples, which may have underestimated the prevalence by missing patent infection. Similarly, malacological and ecological studies, including the bionomics of snail populations and cercariae infection in mollusks, which are key targets in schistosomiasis control, were not conducted. |
|  |  |  | W | 145 |  |  |  |  |  |  |  |  |  |
|  |  |  | M | 105 | 10-15 years old | 3,78 | 4,86 | 43,78% |  |  |  |  |  |
|  |  |  | W | 80 |  |  |  |  |  |  |  |  |  |
| [Nyarko et al. 2018](https://www.ncbi.nlm.nih.gov/pmc/articles/PMC5907294/) | 2016 | Ghana | M | 231 | 9-14 years old |  | 5,20% | 12,10% |  | 0,90% | Prevalence of anemia was 59.9% (mean Hb 11.6 g/dl). The risk of developing anemia was not associated with infection with either parasite. All coinfected children had anemia. Concomitant infection with both parasites may have increased the risk of anemia in coinfected children. The mean hemoglobin concentration of the study population was 11.6 ± 1.5 g/dl. | NA |  |
|  |  |  | W | 173 |  |  | 4,30% | 13,40% |  | 1,20% |  |  |  |
| [Obi et al. 1996](https://cir.nii.ac.jp/crid/1050845764174951552) |  | Nigeria | M | 163 | 5-50 years old |  | 39,88% | 53,99% |  | 20,24% | NA | NA |  |
|  |  |  | W | 105 |  |  | 37,14% | 51,43% |  | 22,86% |  |  |  |
| [Oboth et al. 2019](https://bmcinfectdis.biomedcentral.com/articles/10.1186/s12879-019-3939-x) | 2017-2018 | Uganda | M/W | 74 | <5 years old | 2,50% |  | 52,70% |  | 0,21% | Prevalence of anemia in children aged 6-59 months was 64.9%. Mono-infection (21.3%) and co-infection (0%). Higher prevalence of anemia (56.1%) for plasmodium infection. It is clear that the highest prevalence of anemia was among uninfected children, i.e., 24.1%. | No evidence of malnutrition in 100% of children so median MUAC was 18.8 cm | Small sample size and extremely low helminth prevalence. |
|  |  |  |  | 402 | 5-14 years old | 4,90% |  | 55,70% |  |  |  |  |  |
|  |  |  | M | 256 | <5-14 years old | 5,03% |  | 25,80% |  | 0,21% |  |  |  |
|  |  |  | W | 220 |  | 4,06% |  | 29,20% |  |  |  |  |  |
| [Oladele et al. 2014](http://repository.ui.edu.ng/bitstream/123456789/1134/1/%2845%29%20ui_art_oladele_subtle_2014.pdf) | 2012 | Nigeria | M | 104 | 5->16 years old |  | 29,81% | 28,85% |  | 10,58% | 3.4% of anemic individuals in co-infected individuals, 5% for schistosomiasis infection and 16.4% for plasmodium infection. | Coinfection of malaria and schistosomiasis has been associated with underappreciated subtle morbidity that includes malnutrition. |  |
|  |  |  | W | 136 |  |  | 20,59% | 27,21% |  | 6,18% |  |  |  |
| [Olayinka et al. 2020](https://www.researchgate.net/publication/341651631_Co-infection_of_schistosomiasis_malaria_HBV_and_HIV_among_adults_living_in_Eggua_Community_Ogun_State_Nigeria) | 2015-2017 | Nigeria | M | 167 | 20-60+ years old |  | 22,16% | 16,17% |  | 2,46% | NA | NA |  |
|  |  |  | W | 280 |  |  | 15,00% | 15,00% |  |  |  |  |  |
| [Orish et al. 2019](https://academic.oup.com/ofid/article/6/4/ofz153/5419201) | 2016 | Ghana | M | 249 | 6-14 years old | 0,18% | 12,85% | 71,08% |  | 11,24% | Prevalence of anemia was 18.54%. Prevalence of plasmodium anemia was 18.75%, schistosoma 0% and 36.96% for coninfection. | NA | A major limitation of the current study is that participants provided only a single stool and urine sample, which may have contributed to low levels of intestinal parasites and S. haematobium. However, the low level of intestinal parasites among schoolchildren may reflect the actual situation in the region at the time of this study, which could be attributed to periodic deworming exercises. |
|  |  |  | W | 301 |  |  | 7,97% | 68,11% |  | 5,98% |  |  |  |
| [Raso et al. 2004](https://academic.oup.com/ije/article/33/5/1092/623977) | 2002 | Ivory Coast | M | 249 | 5 days-91 years old | 41,40% |  | 77,50% |  | NA | NA | NA | The extent of very high polyparasitism in this village could be even higher. It is conceivable that mild infections were missed; therefore, the impact on morbidity and comorbidity of these undetected infections is likely to be low. It would be desirable to have a tool with high sensitivity and specificity for accurate diagnosis of parasitic infections of multiple species, which would improve our understanding of the interactions between different parasites. |
|  |  |  | W | 251 |  | 38,30% |  | 75,30% |  |  |  |  |  |
| [Righetti et al. 2012](https://www.ncbi.nlm.nih.gov/pmc/articles/PMC3486899/) | 2010 | Ivory Coast | M/W | 147 | 6-8 years old |  | 10,20% | 78,20% |  | 8,80% | Coinfected children had less anemia. Higher anemia for infection*Plasmodium* | Vitamin A and iron deficiency associated with infection*P. facliparum* |  |
|  |  |  | W | 82 | 15-25 years old |  | 22,00% | 36,60% |  | 8,50% |  |  |  |
|  |  |  | M/W | 95 | 6-23 months |  | 2,10% | 45,30% |  | 1,10% |  |  |  |
| [Salim et al. 2015](https://www.ncbi.nlm.nih.gov/pmc/articles/PMC4383605/) | 2011-2012 | Tanzania | M | 494 | 2 months-9 years |  | 0,40% | 13,10% |  | 0,20% | NA | NA | These children may also be exposed to other environmental risk factors or behave differently in terms of sanitation and hygiene. These were not assessed in the current study, which represents a definitive limitation. |
|  |  |  | W | 498 |  |  |  |  |  |  |  |  |  |
| [Sangweme et al. 2010](https://www.ncbi.nlm.nih.gov/pmc/articles/PMC2976682/) | 2004-2005 | Zimbabwe | M | 485 | 6-17 years old | 21,65% | 64,54% | 25,00% |  | 30,70% | Schistosome infection itself did not cause a significant decrease in mean Hb levels, and malaria infection had the greatest impact on Hb levels. 63% anemia for coinfection, 64% for malaria infection, and 40% for schistosome infection. 38% anemia for uninfected. | NA |  |
|  |  |  | W |  |  |  |  |  |  |  |  |  |  |
| [Sokhna et al. 2004](https://www.ncbi.nlm.nih.gov/pmc/articles/PMC538284/) | 1998-1999 | Senegal |  | 511 | 6-15 years old | 65,75% | 2,70% | 18,98% |  | P+SM= 13.31% | NA | NA |  |
| [Sumbele et al. 2021](https://www.ncbi.nlm.nih.gov/pmc/articles/PMC8152139/) | 2015 | Cameroon | M | 298 | 4-14 years old |  | 20,47% | 23,15% |  | 6,04% | Anemia prevalent in 74.4% of cases. 17.3% anemia for plasmodium monoinfection, 17.7% anemia for schistosome monoinfection and 9.1% anemia in coinfection. 74% prevalence of anemia in women, 74.8% in men. Although the high prevalence of anemia in children aged 4 to 9 years may be related to the high parasite densities of P. falciparum and S. haematobium and the observed microcytosis, the attributable risk of anemia associated with these infections in the population remains low. Nevertheless, the results of the multivariate analysis affirm a negative interaction between the hemoglobin level of malaria parasites and S. haematobium as well as an association between hemoglobin and the linear growth index. Although anemia is one of the morbidity markers associated with schistosomiasis, even though it may be masked by anemia resulting from malaria, the prevalence of anemia in children co-infected with P. falciparum and S. haematobium was very high. | Malnutrition was prevalent in 25.9% of cases. Malnutrition was prevalent in 10.2% of cases with plasmodium monoinfection, 17.2% with schistosoma monoinfection, and 7% with coinfection. Five percent of the sample was underweight, with 20% with plasmodium, 16.7% with schistosoma, and 6.7% with coinfection. The prevalence of malnutrition, underweight, and stunting varied considerably by sex and age, with a significantly higher frequency of moderate and severe stunting in males and children aged 10 to 14 years. | Environmental variations within different localities may be important, explaining the disproportionate distribution of infections across localities, with most co-infections observed in Likoko locality. Limitations of the cross-sectional nature of the design which did not provide changes in burden and morbidities following control measures that could have been captured in a longitudinal study. |
|  |  |  | W | 308 |  |  | 28,57% | 25,64% |  | 10,39% |  |  |  |
| [Sumbele et al. 2021](https://idpjournal.biomedcentral.com/articles/10.1186/s40249-021-00802-x) | 2015 | Cameroon | M | 319 | 4-14 years old |  | 25,08% | 24,92% |  | 6,58% | 74.9% of men are anemic | The prevalence of stunting was 23.7% with a significantly higher prevalence in men and children 11–14 years. 26.5% of malnutrition was observed. 27.3% of malnutrition was associated with S. haematobium infection. | However, the use of a single stool and urine sample for the detection of helminth infection is a limitation of the study as it may have led to an underestimation of the prevalence of polyparasitism as well as the intensities of infections given the variation in daily egg excretion of some of these parasites. Other intestinal parasites may not be detected due to the limited sensitivity of the Kato-Katz technique in detecting other parasites. |
|  |  |  | W | 319 |  |  |  |  |  | 10,97% | 73.7% of women are anemic |  |  |
| [Tay et al. 2017](https://www.ncbi.nlm.nih.gov/pmc/articles/PMC5217638/) | 2012 | Ghana | W pregnant | 62 | 15-19 years old | 4,84% | 6,45% | 29,03% |  | 4% co-infection with all helminths (in addition to *S. mansoni* And*S. haematobium*) | 66.4% of women were anemic. The prevalence of anemia associated with parasitic infections was 49.6%. Plasmodium was significantly associated with anemia (13.3%). Similarly, the presence of *S. haematobium* (3.7%) and*S. mansoni* (5.3%)) were significantly associated with anemia during pregnancy. Anemia was predominant in pregnant women in the age group of 20-29 years, 37.9% (142), and the least dominant occurred in mothers ≥ 40 years, 1.6% (6). | NA | The age groups are not balanced and may therefore bias the results (e.g., higher anemia in 20-29 years (larger group) and lower in ≥ 40 years (smaller group)). This study did not consider data on socioeconomic, behavioral, or environmental factors for analysis. Therefore, potential risk factors for parasitic infections in the study area were not addressed. |
|  |  |  |  | 216 | 20-29 years old | 10,65% | 4,63% | 14,81% |  |  |  |  |  |
|  |  |  |  | 87 | 30-39 years old | 1,15% | 3,45% | 12,64% |  |  |  |  |  |
|  |  |  |  | 10 | 40-49 years old | 10,00% | 0,00% | 10,00% |  |  |  |  |  |
| [Tine et al. 2011](https://www.sciencedirect.com/science/article/pii/S0399077X10002684) | NA | Senegal | M | 68 | 5-15 years old |  | 45,83% | 99.2% (selection of people infected with malaria) |  | 46,22% | NA | NA | Sample too small. |
|  |  |  | W | 52 |  |  |  |  |  |  |  |  |  |
| [Tuasha et al. 2019](https://bmcinfectdis.biomedcentral.com/articles/10.1186/s12879-019-4290-y) | 2009-2010 | Ethiopia | M | 236 | 5->14 years old | 13,35% |  | 47,31% | 16,62% | 2,34% | The prevalence of anemia was 43.12%. Approximately 48.78% of P. falciparum and 39.44% of P. vivax infected were anemic. Prevalence of anemia due to P. falciparum in children aged 5-12 years (53.57%) and in those aged 12-15 years (78.26%). In the present study, S. mansoni was found to increase the risk of developing anemia. | NA | It was considered difficult to reach a definitive conclusion on the interaction of helminthiasis and malaria based on the cross-sectional studies conducted. This is the limitation of the study and a longitudinal/cohort study design on a mixed infection model would be required for a better understanding of the effects of helminth-malaria parasite parts. |
|  |  |  | W | 191 |  |  |  |  |  |  |  |  |  |
| [Yapi et al. 2014](https://www.ncbi.nlm.nih.gov/pmc/articles/PMC4046940/) | 2011-2012 | Ivory Coast | M | 2713 | 5-16 years old | 5,21% | 5,74% | 64,83% |  | 5,56% | NA | NA | Normally 5104 people were used for the study, but from one infection to another, the number increased from 5246 to 5285. Our study has several limitations that deserve consideration. First, given the scale of the work and limited financial and human resources, we were only able to collect single stool, urine, and blood samples for diagnosis. Clearly, more intensive sampling over several days and the use of a range of diagnostic methods would have resulted in higher prevalence estimates, and therefore higher coinfection rates. Second, S. haematobium was indirectly assessed using dipsticks, and although the method is a useful screening tool, the sensitivity and specificity are imperfect compared to urine filtration, which is considered the “gold standard.” (sensitivity range: 64.8–100%; specificity range: 89.6–93%). Third, the study was conducted during the dry season. This could lead to an underestimation of the prevalence of Plasmodium spp., and therefore an underestimated coinfection rate. Fourth, the malaria parasite assessment methods used in this study have some shortcomings. Although we used two methods for the diagnosis of Plasmodium spp. (RDT and standard microscopy), it is conceivable that a polymerase chain reaction (PCR) approach would have allowed the diagnosis of additional cases. The RDT used in our study only identified P. falciparum, but this was considered acceptable, as this is the predominant malaria species in Côte d'Ivoire and neighboring countries. At low parasitemia levels (e.g., 5 parasites/μl of blood), microscopy lacks sensitivity and requires highly trained technicians to accurately diagnose such infections. |
|  |  |  | W | 2391 |  | 1,92% | 5,57% | 58,47% |  |  |  |  |  |

###### ***Supplementary Table 2. Epidemiological surveys on the co-occurrence of schistosomiasis and malaria infections in Africa.*** *This table presents a summary of cross-sectional studies reporting the prevalence of Schistosoma mansoni, Schistosoma haematobium, Plasmodium falciparum and Plasmodium vivax infections, as well as their co-infections. The data include the characteristics of the populations studied (sample size, age, sex, geographical area), the presence of anaemia or malnutrition, and the main methodological limitations reported by the authors. Taken together, the data highlight the epidemiological variability and contextual factors influencing the association between these parasitic diseases.*
