# Supplementary material for: Over-dispersion in malaria–schistosoma co-infection: insights from a meta-analytical approach and systematic review
Source: Infect Dis Poverty. 2026 Oct 9;15:107. doi: 10.1186/s40249-026-01490-1 (PMC13651093; doi:10.1186/s40249-026-01490-1)
Supplement: Supplementary file 3 [file 40249_2026_1490_MOESM8_ESM.docx]

| **Study** | **Study period** | **Geographic area** | **M/W** | **Age** | **% S. mansoni** | | | **% S. haematobium** | | | **% P. falciparum** | | | **%P. vivax** | | | **%Coinfection** | | | **Ho** | | **Immunological measurements** | **Biochemical measurements** | **Anemia** | **Malnutrition** | **Study design** | **Limitations** |
| --- | --- | --- | --- | --- | --- | --- | --- | --- | --- | --- | --- | --- | --- | --- | --- | --- | --- | --- | --- | --- | --- | --- | --- | --- | --- | --- | --- |
|  |  |  |  |  | n | Pos | % | n | Pos | % | n | Pos | % | n | Pos | % | n | Eff | % | Eff | % |  |  |  |  |  |  |
| [Abebe et al. 2024](https://www.hindawi.com/journals/jtm/2024/9992233/) | 2022 | Ethiopia | H | 34 | 5-44+ years old |  |  |  | 34 | 18 | 52,94% | 34 | 18 | 52,94% |  |  |  | 34 | 18 | 52,94% | 28,03% |  | The study revealed that participants co-infected with malaria and S. mansoni had significantly higher mean ALT and AST values, as well as median creatinine, total bilirubin, and direct bilirubin values than those in the healthy control group. Conversely, participants co-infected with S. mansoni and malaria had significantly lower mean glucose levels and median total protein levels than those in the healthy control group. The number of S. mansoni EPG in stool was positively correlated with biochemical profiles (ALT and AST) and negatively correlated with glucose in participants infected with malaria and S. mansoni. The number of S. mansoni EPG in stool was positively correlated with biochemical profiles (creatinine, total bilirubin, and direct bilirubin) and negatively correlated with total protein in participants with malaria and co-infected with S. mansoni. |  |  | Transversal | Candidates were voluntarily selected because they were co-infected or healthy, so the prevalence results are biased. This study has limitations. It recruited participants aged five years and older, excluding children under five years of age and pregnant women. Furthermore, we did not count parasitic infections using molecular techniques such as polymerase chain reaction; we only determined parasite density using microscopy. |
|  |  |  | F | 36 |  |  |  |  | 36 | 17 | 47,22% | 36 | 17 | 47,22% |  |  |  | 36 | 17 | 47,22% | 22,30% |  |  |  |  |  |  |
| Adedoja et al. 2017 | 2012-2013 | Nigeria | M/F | 309 | 4-15 years old |  |  |  | 309 | 94 | 30,42% | 309 | 76 | 25% |  |  |  | 309 | 62 | 20% | 7,44% | IL-10 genotyping. Here, the SNP -1082G/A in the IL-10 promoter and the GCC haplotype composed of the three polymorphisms studied are associated with malaria and the degree of parasitaemia. Polymorphisms in the IL-10 promoter contribute significantly to the regulation of the clinical progression of malaria infection, probably by modulating IL-10 production. |  | NA | NA | Transversal | The small sample size may be a limitation of this study. |
| [Adukpo et al. 2022](https://www.ncbi.nlm.nih.gov/pmc/articles/PMC9494551/) | 2012-2013 | Nigeria | H | 230 | 5-15 years old |  |  |  | 450 | 157 | 34,89% | 450 | 44 | 10% |  |  |  | 450 | 80 | 18% | 3,41% | Identification of plasma levels of GMZ2-specific IgG and its subclasses. IgG levels in Pf/Sh co-infections were generally lower than those in Pf or the uninfected group, but the difference was not significant. The concentrations of IgG1, IgG2, IgG3, or IgG4 did not differ statistically (p>0.05) in the different groups exposed to S. haematobium infection. There was a positive relationship between S. haematobium load and total IgG levels, but not for IgG1, IgG2, IgG3 and IgG4. |  | NA | NA | Transversal | Our inability to detect any relationship between P. falciparum and helminth densities may depend on the clinical conditions of the study participants, the degree of infection, and the age structure of the study participants. This work, however, has some limitations in our inability to use polymerase chain reaction, a more sensitive technique than microscopy, to assess infection, particularly very mild ones. This could have caused us to miss and misclassify these samples. |
|  |  |  | F | 220 |  |  |  |  |  |  |  |  |  |  |  |  |  |  |  |  |  |  |  |  |  |  |  |
| [Ateba Ngoa et al. 2015](https://www.ncbi.nlm.nih.gov/pmc/articles/PMC4365807/) | 2011 | Gabon | H | 64 | 6-16 years old |  |  |  | 64 | 31 | 48,44% | 64 | 37 | 58% |  |  |  | 64 | 18 | 28% | 28,00% | This study did not observe any effect of S. haematobium on the innate and adaptive cytokine profile of subjects infected with P. falciparum. This study shows that infection with P. falciparum is associated with increased immune reactivity that is not affected by co-infection with S. haematobium. Cytokines measured: Innate: IL1b, IP10, MCP, MIP-1β, TNF, IL6, IL10, IL12, IL13, INFγ, MIP-1α; Adaptive: IL2, IL10, IL13, IL17F, IL4, IL5, INFγ, TNF, IL17A, IL21, IL22 and chemokines: iPC1, iPC2, aPC1, aPC2, aPC3. |  | NA | NA | Transversal | An obvious limitation of the present study is that it is cross-sectional, and it could be argued that it does not provide information on the history of past helminth infections, which are capable of leaving a mark on the host's immune system. For example, it has been shown that the innate immune system is capable of maintaining a ‘memory’ of early exposure to PAMPs through a process called ‘trained immunity,’ which is not addressed in this study (Netea et al. 2011). Another limitation concerns the sample size of the study, which may not be sufficient to detect an effect of helminths on P. falciparum-modulated immune responses. |
|  |  |  | F | 61 |  |  |  |  | 61 | 32 | 52,46% | 61 | 29 | 48% |  |  |  | 61 | 14 | 23% | 24,93% |  |  |  |  |  |  |
| [Ateba Ngoa et al. 2016](https://www.ncbi.nlm.nih.gov/pmc/articles/PMC4973188/) | NA | Gabon | H | 153 | NA |  |  |  | 150 | 112 | 74,67% | 262 | 197 | 75% |  |  |  | 284 | 146 | 51% | 56,66% | The antibody responses measured were for the sexual (Pfs230, Pfs48/45) and asexual antigens of P. falciparum (AMA1, MSP1 and GLURP). When the asexual form of P. falciparum was determined by PCR, higher antibody concentrations were observed in infected subjects compared to uninfected subjects for Pfs230 and AMA1 but not for the other antibodies. There was no apparent association between Pfs230, AMA1, GLURP or MSP1 antibody responses and S. haematobium infection, but there was a significant decrease in Pfs48/45 antibody titres in schistosome-infected subjects compared to uninfected subjects. When the analysis was restricted to subjects not infected with S. haematobium, P. falciparum infection determined by PCR was associated with a significant increase in Pfs48/45 and Pfs230 specific antibodies. This association was not observed when the model was applied to individuals infected with S. haematobium. |  | Average haemoglobin level of 11.2 g/dL for individuals infected with S. haematobium. | NA | Transversal | The study has a cross-sectional design and does not include equipment for the sensitive detection of gametocytes using RNA-based methods. |
|  |  |  | F | 134 |  |  |  |  | 134 | 102 | 76,12% |  |  |  |  |  |  |  |  |  |  |  |  |  |  |  |  |
| [Bustinduy et al. 2015](https://sci-hub.53yu.com/10.4269/ajtmh.14-0444) | 2009 | Kenya - Milalani | H | 95 | 2-19 years old |  |  |  | 191 | 130 | 68,06% | 191 | 33 | 17% |  |  |  | 191 | NA | NA | 11,76% | IL-10 significantly elevated in 13-14 year old children infected with S. haematobium. After adjustment for cofactors, IL-10 levels were higher in S. haematobium-infected children compared to S. haematobium-uninfected children. IL-6 was particularly elevated in S. haematobium-infected children aged 11-12 years (compared to S. haematobium-negative children in the same age group and other groups) and IL-10 higher in S. haematobium-infected children aged 13-14 years. Elevated TNF-α levels were observed in children aged 11-12 years, regardless of their infection status. Plasmodium-infected children had higher IL-6 levels and affected children were younger (5-7 years old). |  | 47% anemia among children in Milalani | 32% of children are stunted and 11% are thin. | Transversal | Current serum cytokine levels measured in cross-sectional images may not accurately reflect the in vivo effects of infection related to different antigenic stimulations. Detection of cytokines at a single time point in the circulation, rather than in local tissue microenvironments, is an inherent limitation of this type of study. The study involved a single cross-sectional image, and therefore, no post-treatment changes could be established to support parasite-specific associations. Exploration of other cytokines not included in this study (i.e., IL-4, IL-5, and IL-13) that are important for understanding the immune response to multiple parasite infections should be incorporated into future research efforts. |
|  |  |  | F | 96 |  |  |  |  |  |  |  |  |  |  |  |  |  |  |  |  |  |  |  |  |  |  |  |
|  |  | Kenya - Vuga | H | 300 |  |  |  |  | 613 | 155 | 25,28% | 613 | 77 | 13% |  |  |  | 613 | NA | NA | 3,17% |  |  | 41% anemia among children in Vuga | 43% of children are stunted and 31% are thin. |  |  |
|  |  |  | F | 313 |  |  |  |  |  |  |  |  |  |  |  |  |  |  |  |  |  |  |  |  |  |  |  |
| [Courtin et al. 2011](https://sci-hub.53yu.com/10.1111/j.1365-3024.2010.01267.x) | 2003 | Senegal | H | 155 | 7-19 years old |  |  |  | 155 | 109 | 70,32% | 305 | 272 | 89% |  |  |  | 234 | 78 | 33% | 58,69% | IgG levels against MSP-1 and GLURP were significantly lower in the S. **haematobium than in the uninfected group. Association between the presence of S. haematobium and the decrease in antibody levels associated either with protection against clinical attacks of P. falciparum (IgG1 to GLURP), or with the control of parasitaemia (IgG1 to MSP-1).**Although not statistically significant, the same trends of decreased cytophilic IgG levels were observed for antibodies to the other antigens tested (AMA-1, MSP-2, and MSP-3). Individuals infected with S. haematobium had significantly higher levels of IL-10 and IFN-γ than uninfected individuals. Individuals infected with P. falciparum had significantly higher levels of IL-10 and IL-6 than uninfected individuals. P. falciparum infection was associated with increased levels of IL-10, and S. haematobium infection was associated with increased levels of IL-10, IFN-γ, and IL-6. Higher circulating levels of IL-10 in the plasma of co-infected individuals were associated with decreased anti-plasmodial IgG responses, particularly those directed against MSP-2. |  | NA | NA | Prospective cohort | Urinary tract infection with schistosomiasis was determined by the detection of S. haematobium eggs using a urine filtration technique and not by PCR or other more precise techniques. |
|  |  |  | F | 79 |  |  |  |  | 79 | 45 | 56,96% |  |  |  |  |  |  |  |  |  |  |  |  |  |  |  |  |
| [Diallo et al. 2010](https://www.ncbi.nlm.nih.gov/pmc/articles/PMC2939900/) | NA | Senegal | M/F | 79 | 7-15 years old |  |  |  | 79 | 39 | 49,37% | 79 | 79 | 100% |  |  |  | 79 | 39 | 49% | 49,37% | **Plasma concentrations of IL-10, TGF, INF γ (but not INF α) were higher in co-infected subjects than in those with a single infection**. **Significant increase in humoral response to Plasmodium antigens in Schistosoma-infected subjects**. **S. haematobium coinfection influenced the humoral immune response against malaria antigens by specifically increasing IgG1 and IgG3 levels** .**However, only the specific IgG1 Ab response to MSP1-19 antigen was significantly increased in coinfected children, suggesting that the influence of coinfection might be antigen dependent.** The MSP1-19 specific IgG3 response was observed in 1/3 of co-infected children but not in Pf mono-infected children. **Helminth infections influence the production of IL-12, IL-13, and TGF-β upon stimulation with both malaria antigens.**In contrast, schizont extract-specific IFN-γ and IL-10 production was significantly higher in the coinfected group, which also had predominant levels of anti-schizont IgG1 and IgG3. MSP1-19-specific IL-10 production was significantly higher in the coinfected group than in Pf monoinfected, as was the specific IgG1 response. |  | NA | NA | Transversal | Patients were selected for the study ONLY if they were infected with Plasmodium (=bias). The influence of the environment, genetic background, or nutritional status cannot be excluded to explain the variation in specific immunity observed in our study. |
| [Diallo et al. 2004](https://www.et-fine.com/10.1111/j.0141-9838.2004.00719.x) | NA | Senegal | M/F | 79 | 7-15 years old |  |  |  | 79 | 39 | 49,37% | 79 | 79 | 100% |  |  |  | 79 | 39 | 49% | 49,37% | Increased production of antifibrogenic INFγ in co-infected children. TNF-α level was not significantly affected by co-infection. Only co-infected adults showed a significant increase in IL-10 and TGF-β levels. Significant increase in plasma concentration of sTNF receptors, sTNF-RI and sTNF-RII in adults. In children, only the plasma level of sTNF-RII was significantly higher in the co-infected group compared to the mono-infected group. Statistical tests showed the absence of correlation between parasitemia and the variation in cytokine levels observed between the Pf/Sh and Pf groups. In contrast to children, the mean IL-10/TNF-α ratio was significantly higher in co-infected adults, **suggesting that high IL-10 levels may be specifically associated with co-infection**malaria, regardless of plasma TNF-α level. |  | NA | NA | Transversal |  |
|  |  |  | M/F | 48 | 30+ years old |  |  |  | 48 | 18 | 37,50% | 48 | 48 | 100% |  |  |  | 48 | 18 | 38% | 37,50% |  |  |  |  |  |  |
| [Lyke et al. 2006](https://www.ncbi.nlm.nih.gov/pmc/articles/PMC1594876/) | 2002-2003 | Mali | H | 295 | 4-14 years old |  |  |  | 295 | 158 | 53,56% | 607 | 505 | 83% |  |  |  | 607 | 252 | 42% | 44,41% | The cytokine serological milieu at baseline and at the time of acute falciparum malaria infection appears to be altered by the presence of underlying schistosomiasis infection. At baseline, Th2 cytokine (IL-4, IL-6, and IL-10) and IFN-γ levels were elevated in Sch(+) children. Our results demonstrated that there were elevated levels of Th2 cytokines, as well as elevated levels of IFN-γ, in children with schistosomiasis compared with uninfected children and suggest that IL-4 and IL-10 may play an important role in modulating the host response to malaria infection. |  |  |  | Transversal |  |
|  |  |  | F | 312 |  |  |  |  | 312 | 166 | 53,20% |  |  |  |  |  |  |  |  |  |  |  |  |  |  |  |  |
| [Lyke et al. 2012](https://www.ncbi.nlm.nih.gov/pmc/articles/PMC3279404/) | 2002-2003 | Mali | H | 30 | 4-14 years old |  |  |  | 30 | 18 | 60,00% | 30 | 22 | 73% |  |  |  | 30 | 10 | 33% | 44,00% | We report the presence of Tregs in both malaria and S. haematobium infections. Our studies provide strong evidence that the presence of Tregs at the time of acute falciparum malaria infection is reduced in the presence of underlying schistosome infection compared to age-matched children without helminthic infection. Children with falciparum malaria alone had higher percentages of Tregs, as well as reduced circulating Th1 cytokines (IFN-γ and IL-2) and higher parasitemias, compared to age-matched children with coinfection. We did not observe an elevation of Tregs as would be expected during schistosome infection. |  | NA | NA | Transversal | The complicated logistics of obtaining PBMCs from the large cohort of children at enrollment precluded sample collection before the malaria transmission season. We did not examine dry-season samples for low-level parasitemia to explain why Tregs may continue to exert suppressive effects on effector responses. |
|  |  |  | F | 19 |  |  |  |  | 19 | 8 | 42,10% | 19 | 19 | 100% |  |  |  | 19 | 8 | 42% | 42,10% |  |  |  |  |  |  |
| [Morenikeji et al. 2016](https://www.ncbi.nlm.nih.gov/pmc/articles/PMC4894265/) | 2014 | Nigeria | M/F | 332 | 5-19 years old |  |  |  | 332 | 92 | 27,71% | 332 | 136 | 41% |  |  |  | 332 | 31 | 9% | 11,35% | The anti-Schistosoma antibody response depended on the density of Plasmodium spp. Although coinfection with malaria parasites appears to increase anti-Schistosoma IgG, decreasing malaria parasitemia appears to increase antibody production. Conversely, increasing the intensity of S. haematobium infection has been reported to result in decreased levels of the cytophilic subclasses IgG1, IgG2, IgG3, and IgG4 against Plasmodium falciparum. |  | NA | NA | Transversal | Three species of Plasmodium were considered in the study, and there are no specific data for P. falciparum. The anti-Schistosoma antibodies recorded among individuals diagnosed with Plasmodium spp. infection only could indicate the presence of false negative tests for S. haematobium in some individuals, particularly in cases of very mild infection. Another possible reason is the persistence of humoral circulation of anti-Schistosoma antibodies in individuals treated for Schistosoma infection who were previously co-infected with malaria parasites. The inability of the IgG enzyme-linked immunosorbent assay (ELISA) to distinguish active Schistosoma infection from the chronic type is one of the main obstacles to its application. Cross-reactivity between Schistosoma and malaria parasite antigens could also account for the presence of anti-Schistosoma antibodies in individuals infected only with Plasmodium spp. Limitations of this study include the use of non-molecular methods for infection detection, which could compromise the true infection status. There is also a need to link the influence of age, sex, and specific malaria parasite species to the antibody response in single and co-infections. |
| [Mutapi et al. 2007](https://www.scielo.br/j/mioc/a/WJc9TfxKgCBm6jZ76tG3zyH/?lang=en) |  | Zimbabwe |  | 42 | 6-14 years old |  |  |  |  |  | 42,80% |  |  | 26% |  |  |  |  |  |  | 11,13% | At low schistosome infection intensities, levels of cytophilic IgG1, IgG3, and IgG4 isotypes against total malaria extract are elevated and decrease significantly with increasing schistosome infection intensity. In addition, levels of IgG2, IgG3, and IgG4 against the same antigen were positively correlated with levels of the same isotypes against soluble schistosome egg antigen. CD4 + TEM populations are decreased in schistosome-infected individuals compared to uninfected individuals. No association between schistosome infection intensity/specific antibodies and responses against MSP3b. The only association with Glurp R0 responses was a significant negative correlation between IgG2 to schistosome-SEA and Glurp R0.**Six months after treatment of schistosome, there was a significant increase in IgG4 levels against MSP3b and IgM against Glurp R0 as well as a significant decrease in IgG4 levels against Glurp R0, but not in crude, total extracted antigen.** |  | NA | NA | Prospective cohort | Sample too small. |
| [Naus et al. 2003](https://academic.oup.com/jid/article/187/8/1272/862372?login=false) | NA | Kenya - Immigrants | H | 91 | 5-59 years old | NA | NA | NA | NA | NA | NA | NA | NA | NA | NA | NA | NA | NA | NA | NA | NA | Strong correlation between malaria- and schistosome-specific IgG3 responses. Cross-reactivity between IgG3 directed against P. falciparum MSP1 antigens and the adult S. mansoni worm in individuals exposed to both parasites. The anti-Pfs IgG3 response in the immigrant population was higher than that of locals in the region. Cross-reactivity between the P. falciparum schizont and the S. mansoni worm in diverse human populations living in areas where the two infections are co-endemic. This cross-reactivity is largely limited to the IgG3 isotype response. |  | NA | NA | Prospective cohort |  |
|  |  |  | F | 93 |  |  |  |  |  |  |  |  |  |  |  |  |  |  |  |  |  |  |  |  |  |  |  |
|  |  | Kenya - Locals | H | 87 |  | NA | NA | NA | NA | NA | NA | NA | NA | NA | NA | NA | NA | NA | NA | NA | NA |  |  |  |  |  |  |
|  |  |  | F | 148 |  |  |  |  |  |  |  |  |  |  |  |  |  |  |  |  |  |  |  |  |  |  |  |
|  |  | Uganda | H | 148 | 7-50 years old | NA | NA | NA | NA | NA | NA | NA | NA | NA | NA | NA | NA | NA | NA | NA | NA |  |  |  |  |  |  |
|  |  |  | F | 129 |  |  |  |  |  |  |  |  |  |  |  |  |  |  |  |  |  |  |  |  |  |  |  |
|  |  | Sudan | H | 24 | 14-75 years old | NA | NA | NA | NA | NA | NA | NA | NA | NA | NA | NA | NA | NA | NA | NA | NA |  |  |  |  |  |  |
|  |  |  | F | 22 |  |  |  |  |  |  |  |  |  |  |  |  |  |  |  |  |  |  |  |  |  |  |  |
| [Nouatin et al. 2021](https://www.ncbi.nlm.nih.gov/pmc/articles/PMC8195366/) | 2015 | Gabon | H | 39 | 22-25 years old |  |  |  | 39 | 14 | 35,90% | 39 | 12 | 31% |  |  |  | 39 | NA | NA | 11,05% | The results show that**Helminth species may differentially affect the specific immune response following administration of a malaria vaccine candidate**Participants infected with S. hematobium had higher GMZ2-specific IgG at post-first D84 vaccination. Of note, S. haematobium infection had no significant effect on antibody responses to MSP3 or GLURP, the two antigens combined in GMZ2, although the anti-GLURP IgG concentration level was higher for S. hematobium infection compared to uninfected, but this difference was not statistically significant. |  | NA | NA | Transversal | The sample size is too small, precluding further in-depth statistical testing. There is too little detailed data on S. haematobium and none on coinfection with Plasmodium. Furthermore, the study was conducted exclusively on men, which prevents extrapolation of any conclusions to the general population. The observational and exploratory nature of the study also means that causality cannot be easily addressed. Another possible limitation is the CHMI, as it has never been used before in this setting. Furthermore, the fact that not all available plasma at different time points was analyzed and that the diagnosis of helminths was not made between D0 and D84 weakens the results, because this would allow us to verify whether these same observations are made at other time points. |
| [Oboh-Imafidon et al. 2023](https://www.ncbi.nlm.nih.gov/pmc/articles/PMC10486024/) | 2015 | Nigeria | H | 77 | 10.97 years old |  |  |  | 144 | 112 | 77,78% | 144 | 64 | 44,44% |  |  |  | 144 | 64 | 44,44% | 44,44% |  |  | NA | NA | Transversal | Our study is limited by the small number of patients that may have contributed to the currently observed results. As a single-center study, these results may not be generalizable or representative enough to be applicable to other areas where malaria and schistosomiasis are coinfected in Africa and elsewhere. |
|  |  |  | F | 67 |  |  |  |  |  |  |  |  |  |  |  |  |  |  |  |  |  |  |  |  |  |  |  |
| [Remoue et al. 2003](https://academic.oup.com/trstmh/article-abstract/97/3/361/1941328?redirectedFrom=fulltext) | 1996 | Senegal | M/F | 38 | 7-15 years old |  |  |  | 38 | 38 | 100,00% | 38 | 23 | 60,52% |  |  |  | 38 | 23 | 60,52% | 60,52% | The low levels of specific IgM, IgG1, IgG2, IgG4, and IgA responses were similar in both groups of infected children. In contrast, anti-Sh28GST IgG3 and IgE antibody levels were significantly higher in coinfected children than in children infected only with S. haematobium. Furthermore, the specific IgE response appeared to be induced only in coinfected children (except for one child infected only with S. haematobium). Anti-SEA IgG3 and IgE responses were also significantly higher in coinfected children, in contrast to other isotype responses. IFN-γ and IL-10 levels were significantly higher in coinfected children than in children infected only with S. haematobium. The median IL-10/TNF-α ratio was higher in coinfected children. TNF-RI and TNF-RII levels were predominant in co-infected children compared to S. haematobium mono-infected children and only plasma TNF-RII level was significantly higher in co-infected children. |  |  |  | Transversal | Sample size too small. All participants are selected because they are infected with schistosome. |
| [Sangweme et al. 2010](https://www.ncbi.nlm.nih.gov/pmc/articles/PMC2976682/) | 2004-2005 | Zimbabwe | M/F | 485 | 6-17 years old | 485 | 105 | 21,65% | 485 | 313 | 64,54% | 485 | 138 | 28,45% |  |  |  | 485 | 91 | 18,76% | 26,16% | In general, total specific IgG activity and levels showed a progressive increase from T0 to T12, reflecting a likely increase in malaria-specific antibody levels. Plasma from malaria-infected subjects showed higher antimalarial antibody activity in response to malaria antigens. At T0 in the entire study population, IgG levels to MSP-1 were also higher in malaria-infected subjects than in their malaria-free counterparts. There was a trend for elevated IgG3 isotype antibodies to MSP-1 and AMA-1 in the coinfected group. Antibody levels to malaria antigens were similar between the two groups at T0. Plasma growth inhibition activity was higher in children infected with malaria alone than in coinfected children, although antibody levels were comparable for both groups. |  | Schistosome infection itself did not cause a significant decrease in mean Hb levels, and malaria infection had the greatest impact on Hb levels. Differences in Hb levels were significant between uninfected and Plasmodium-infected, uninfected, and coinfected children, but not between uninfected and Schistosoma-infected children. At T0, 38% of children in the uninfected group, 40% in the Schistosoma-infected group, 64% in the Plasmodium-infected group, and 63% in the coinfected group were classified as anemic. |  | Prospective cohort | The figures for schistosomiasis prevalence are not really clear. Perhaps seasonal fluctuations in mean plasma GIA depending on the malaria season should have been taken into account. |
| [Santano et al. 2021](https://www.ncbi.nlm.nih.gov/pmc/articles/PMC8653825/) | NA | Mozambique | H | 311 | NA |  |  |  | 311 | 195 | 27,27% | 311 | 279 | 89,71% |  |  |  | 715 | NA | NA | 24,83% | Antigen-specific IgG and total IgE responses were higher in the coinfection group than in the monoinfection group. Coinfection was associated with increased Schistosoma spp antigen-specific IgG and IgE responses compared with the monoinfection group. In our linear models, IgE was positively associated with P. falciparum-specific IgG levels, which have been shown to reflect exposure rather than protection. |  |  |  | Transversal | No detailed data on schistosome prevalence but on helminths in general. A caveat of this study is the inability to determine causality due to its cross-sectional nature. A longitudinal design, on the other hand, would be more appropriate to determine whether individuals with P. falciparum or helminths are more susceptible to subsequent infections. Furthermore, this study only included asymptomatic malaria cases, which may hamper the detection of important associations with disease severity and mask the true burden of co-infections. An additional limitation is the pooling of all helminth species due to the small sample size of the species-level groups, which may result in masked effects of individual species. Finally, we could not assess whether there was some cross-reactivity between antibodies to the helminth and P. falciparum antigens, which is unlikely but could have affected the increased antibody levels observed in the co-position/coinfection group. |
|  |  |  | F | 404 |  |  |  |  | 404 |  |  | 404 | 372 | 92,08% |  |  |  |  |  |  |  |  |  |  |  |  |  |
| [Tokplonou et al. 2020](https://onlinelibrary.wiley.com/doi/10.1111/pim.12702) | 2013 | Benign | H | 178 | 4-8 years old |  |  |  | 178 | 59 | 33,15% | 268 | 121 | 45,15% |  |  |  | 268 | 38 | 14,18% | 15,16% | Sh-infected children have higher concentrations of various IgG isotypes directed against MSP3 and GLURP antigens than uninfected children. No differences in IL-10 levels and regulatory T cell frequencies in Sh-infected and uninfected children were observed in the present study. High concentrations of antimalarial antibodies observed in Sh-infected individuals. High level of antibodies in co-infected individuals. Co-infected children had a lower frequency of T cellsγδ than those mono-infected with Pf. Higher levels of IFNγ in Pf-infected individuals. LILRB2 expression was lower on the surface of neutrophils from co-infected children than from uninfected ones. Our study demonstrates that the presence of Sh modulates host immune responses directed against Pf. |  | 89% of children had at least moderate anemia and 25% of them had hemoglobinopathy. 87% of children infected with S. haematobium were anemic. |  | Prospective cohort | Helminth diagnosis was not routinely performed monthly like malaria diagnosis, and therefore it was not possible to know precisely when children were infected during follow-up. Therefore, some Pf parasitemias included in the calculation of mean parasite density during follow-up in the Sh-positive group were likely detected when children were not yet infected with Sh. Such misclassification may increase the mean Pf parasitemias in the Sh-positive group, making it more difficult to identify associations between Sh status and mean Pf parasite density. We did not observe in our study the immune regulatory pattern usually reported during helminth infection. |
|  |  |  | F | 90 |  |  |  |  | 90 | 31 | 34,44% |  |  |  |  |  |  |  |  |  |  |  |  |  |  |  |  |
| [Wilson et al. 2008](https://www.ncbi.nlm.nih.gov/pmc/articles/PMC2346678/) | NA | Kenya | M/F | 79 | 4-17 years old |  |  |  | 79 | 79 | 100,00% | 79 | NA | 74,70% |  |  |  | 79 | NA | 74,70% | 74,70% | Coinfections lead to decreased Th2 cytokine responses to egg antigens, while they lead to increased TNF-α production. Levels of SEA-specific Th2 responses were low. Third principal component scores, substantially explained by variation in SEA-specific Th2 cytokine responses IL-4, IL-13, and IL-5, as well as SEA-specific IFNγ, were negatively correlated with S. mansoni egg counts, indicating that SEA-specific Th2 responses are downregulated depending on the intensity of infection. SWA-specific Th2 responses were both greater than SEA-specific Th2 responses and unrelated to the intensity of S. mansoni infection. SEA-specific Th2 responses were also lower in coinfected children. SEA induced particularly high levels of TNF-α. |  |  |  | Prospective cohort | All selected children had S. mansoni infection. Sample size too small. It was not possible to confirm that hepatosplenomegaly is associated with a high level of pro-inflammatory reactivity to schistosome antigens. |

***Supplementary Table 3. Functional studies on the immunological and biochemical impact of schistosomiasis–malaria co-infections.*** *This table summarises functional studies conducted in Africa exploring the immunological, biochemical and clinical consequences of co-infections between Schistosoma spp. and Plasmodium spp. The data include the characteristics of the populations studied, the prevalence of single and combined infections, and the observed effects on immune responses (cytokines, antibodies, regulatory cells), biochemical parameters (ALT, AST, bilirubin, glucose, proteins), and clinical markers (anaemia, malnutrition). The reported studies highlight complex interactions between the two parasitic diseases, which may modulate susceptibility, vaccine response, and the clinical severity of malaria.*
