# Supplementary material for: Over-dispersion in malaria–schistosoma co-infection: insights from a meta-analytical approach and systematic review
Source: Infect Dis Poverty. 2026 Oct 9;15:107. doi: 10.1186/s40249-026-01490-1 (PMC13651093; doi:10.1186/s40249-026-01490-1)
Supplement: Supplementary file 4 [file 40249_2026_1490_MOESM9_ESM.docx]

Appendix S1

Appendix S2

Appendix S3
